# Deciphering the mechanisms of antibacterial and antibiofilm potential of phenolic compounds against *Serratia marcescens*

**DOI:** 10.1186/s40643-025-00988-0

**Published:** 2025-12-08

**Authors:** Pooja Pandey, Sirisha L. Vavilala

**Affiliations:** School of Biological Sciences, UM DAE Centre for Excellence in Basic Sciences, University of Mumbai, Vidyanagari, Kalina Campus, Santacruz East, Mumbai, 400098 India

**Keywords:** Coumaric acid, Syringic acid, Apoptosis-like cell death, Biofilm inhibition, Biofilm eradication, Quorum sensing, Swimming and swarming motility, Virulence factors

## Abstract

Biofilm formation significantly contributes to antibiotic resistance in *Serratia marcescens*, a multidrug-resistant pathogen implicated in persistent respiratory infections. This study investigates the antibacterial and anti-biofilm potential of two plant-derived phenolic acids—coumaric acid and syringic acid—against *S. marcescens*. Both compounds demonstrated strong inhibitory activity against planktonic cells, exhibiting low MIC values. They induced membrane permeabilization and leakage, leading to cell death, and triggered externalization of phosphatidylserine—hallmarks of apoptosis-like death in bacteria. Coumaric and syringic acids markedly inhibited biofilm formation by 70% and 80%, respectively, and efficiently disrupted mature biofilms by degrading extracellular DNA (eDNA), a key structural component of the EPS matrix. The eDNA-binding assays revealed strong interactions between the compounds and eDNA, with reduced fluorescence confirming their destabilizing effects on biofilm architecture. Scanning electron microscopy (SEM) and confocal laser scanning microscopy (CLSM) further corroborated these findings, showing disrupted biofilm structure and a predominance of dead cells in treated groups. In addition, both compounds significantly attenuated quorum sensing (QS)-regulated virulence factors and inhibited bacterial motility, including swimming and swarming, critical for biofilm propagation. These combined antibacterial, anti-biofilm, and anti-virulence effects suggest that coumaric and syringic acids act through a comprehensive mechanism disrupting both cellular integrity and intercellular communication pathways essential for *S. marcescens* pathogenicity. Together, these findings highlight the potential of coumaric and syringic acids as promising phytochemicals for the development of novel anti-biofilm therapeutics targeting antibiotic-resistant *S. marcescens* infections.

**Graphical abstract:**

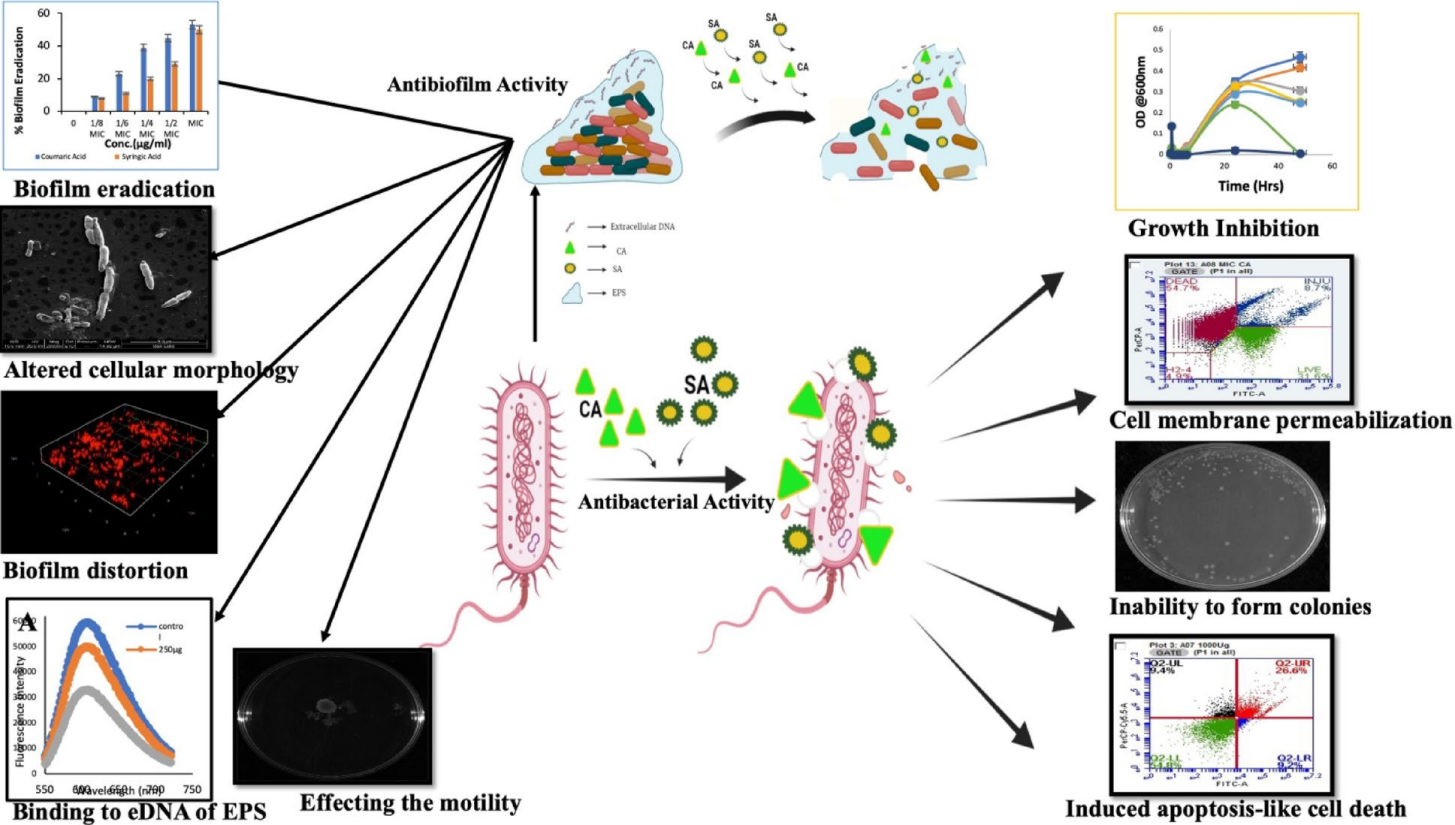

**Supplementary Information:**

The online version contains supplementary material available at 10.1186/s40643-025-00988-0.

## Introduction

Antimicrobial resistance (AMR) is recognized as one of the most critical global health threats of the twenty-first century. While the discovery and early development of antibiotics revolutionized medicine and initially curbed resistance, the pace of novel antibiotic discovery has drastically slowed since the late 1960s, with only a few new antibiotic classes emerging in recent decade. Multidrug-resistant (MDR) bacteria increasingly render standard therapies ineffective, leading to prolonged illness, higher treatment costs, and escalating mortality, with disproportionate effects in vulnerable populations and resource-limited regions (Aminov 2010; Nikaido 2009; Hernando-Amado et al. 2019; Salam et al. 2023a; Carvalho et al. 2022; Van Boeckel et al. 2019). According to the World Health Organization, AMR currently accounts for an estimated 1.27 million deaths annually, with projections warning upto 10 million deaths per year by 2050 if decisive interventions are not implemented (ARC Global and burden of bacterial antimicrobial resistance in 2019; Salam et al. 2023b). The underlying mechanisms driving resistance include target modification, efflux pumps, immunity and bypass pathways, enzyme-mediated drug destruction, and biofilm formation (Sabtu et al. 2015; Wright 2010). Among these, biofilm formation represents a key contributor to chronic and persistent infections. It increases bacterial resistance to antibiotics, acidic environments, and nutrient scarcity. This intrinsic resistance arises from the synergistic interplay of acquired and adaptive mechanisms within biofilms, creating a protective niche that shields resistant bacteria (Yi et al. 2020). Moreover, biofilms serve as dynamic hubs for horizontal gene transfer, where dense microbial communities and mobile genetic elements facilitate the exchange of resistance and virulence determinants. Plasmid conjugation, transposon mobilisation, and insertion sequences promote the dissemination of resistance and virulence traits, facilitating the coexistence of both commensal and highly virulent strains across species boundaries within polymicrobial biofilms (Costerton et al. 2005).

The ESKAPE pathogens (*Enterococcus faecium*, *Staphylococcus aureus*, *Klebsiella pneumoniae*, *Acinetobacter baumannii*, *Pseudomonas aeruginosa*, and members of the *Enterobacteriaceae* family) account for the majority of hospital-acquired infections due to their remarkable ability to evade antimicrobial therapies. Within this group, *S. marcescens*, a member of *Enterobacteriaceae*, has emerged as a formidable opportunistic pathogen, exhibiting intrinsic resistance mechanisms, robust biofilm formation, and rising multidrug resistance, substantially hindering the effective treatment of hospital-acquired infections such as pneumonia, bloodstream infections, and urinary tract infections (Denissen et al. 2022).

*Serratia marcescens*, a Gram-negative bacillus of the *Enterobacteriaceae* family, is a well-recognized cause of hospital-acquired infections, including septicaemia, meningitis, urinary tract infections, wound infections, and respiratory and genital tract infections (Chen et al. 2003; Hejazi and Falkiner 1997). Over the past two decades, it has emerged as a major nosocomial pathogen with escalating multidrug resistance. Global surveillance reports indicate that 40–60% of clinical isolates exhibit resistance to third-generation cephalosporins and aminoglycosides, with resistance to carbapenems also reported in certain regions. This multidrug-resistant phenotype is mediated by multiple mechanisms, including β-lactamase production (notably AmpC), efflux pump activation, and reduced outer membrane permeability, which collectively enable survival against diverse antibiotic classes (Sethupathy et al. 2020a). Beyond these mechanisms, S. marcescens exploits quorum sensing (QS) as a critical regulatory system controlling virulence, biofilm formation, and antibiotic resistance. The SmaIR QS system, homologous to the canonical LuxIR system, orchestrates these processes, with SmaI synthesizing N-acyl homoserine lactones (AHLs) and SmaR functioning as the receptor-regulator modulating target gene expression (Van Houdt et al. 2007).

This Gram-negative pathogen employs an N-acyl homoserine lactone (AHL)-mediated QS system—primarily the SmaI/SmaR circuit—to modulate the expression of genes involved in the production of key virulence factors such as proteases, lipases, hemolysin, and the red pigment prodigiosin, all of which contribute to its pathogenicity and persistence in host environments (Eberl et al. 1996; Horng et al. 2002; Coulthurst et al. 2006; Van et al. 2007; Gokhale et al. 2024). QS also orchestrates the transition from planktonic to sessile lifestyles by promoting extracellular polymeric substance (EPS) synthesis and structural maturation of biofilms, enhancing resistance to both host defences and conventional antibiotics (Ray et al. 2017; Sethupathy et al. 2020b). The biofilm mode of growth creates diffusion barriers and induces physiological heterogeneity, contributing to phenotypic tolerance and the upregulation of efflux pumps and stress response genes under QS control (Stewart and Costerton 2001; Høiby et al. 2010; Stewart 2002; Brackman et al. 2011). Given its central role in pathogenicity and multidrug resistance, quorum sensing (QS) represents a promising anti-virulence target. Inhibiting QS offers a strategy to attenuate *S. marcescens* pathogenicity without imposing the selective pressure associated with conventional antibiotics. The organism’s alarming resistance to nearly all major antimicrobial classes highlights the urgent need for alternative therapeutic approaches. Natural compounds are particularly attractive as QS inhibitors, as they often provide potent efficacy with reduced toxicity and fewer side effects.

Phenolic acids are multifunctional plant-derived compounds with broad pharmacological activities, including antioxidant, anti-inflammatory, and antimicrobial effects. Their ability to modulate cellular processes such as oxidative stress, proliferation, and apoptosis has positioned them as promising candidates for therapeutic applications. Importantly, many phenolic acids also interfere with bacterial quorum sensing (QS) and biofilm formation, offering a natural anti-virulence strategy without exerting strong selective pressure for resistance. Among them, p-coumaric acid (CA) and syringic acid (SA) are particularly attractive due to their abundance in dietary plants and well-documented bioactivities. CA, a major component of cereal grain cell walls, exhibits strong antibacterial and antioxidant activities, making it a potential alternative to synthetic antimicrobials. SA, widely distributed in fruits and vegetables, possesses antioxidant, anti-inflammatory, and antibacterial properties attributed to its methoxy substitutions, which enhance its bioactivity. These phytochemicals disrupt N-acyl homoserine lactone (AHL)-mediated signalling pathways, inhibit biofilm maturation, and downregulate QS-controlled virulence gene expression in multiple Gram-negative bacteria. Although their molecular mechanisms in *S. marcescens* QS regulation remain insufficiently characterized, these compounds are postulated to interfere with QS signal transduction cascades and transcriptional regulators, thereby attenuating pathogenic phenotypes and biofilm-associated persistence. Given their natural origin, safety profile, multi-targeted bioactivity, and low cytotoxicity, coumaric acid and syringic acid represent promising candidates for the development of novel anti-virulence therapeutics aimed at mitigating antimicrobial resistance (Pandey et al. 2024a; Silva et al. 2016; Tajani et al. 2023; Gutiérrez-Barranquero et al. 2015; Zhang et al. 2018; Yazıcı et al. 2023; Chen et al. 2020; Alain et al. 2022; Beddiar et al. 2021).

This study aimed to investigate the antibacterial and antibiofilm activities of the phenolic acids coumaric acid and syringic acid against *S. marcescens*. A multidisciplinary approach combining biochemical assays, cellular studies, and advanced imaging was employed to examine how these compounds interfere with bacterial growth, virulence factors, and biofilm stability. Together, these complementary methodologies provide a comprehensive mechanistic understanding of how these natural compounds attenuate pathogenicity and destabilize mature biofilms of *S. marcescens*.

## Materials and methods

Coumaric acid and Syringic acid were purchased from Sigma-Aldrich and used in their original form. Working stocks were made from the master stock (10 mg mL^−1)^ and used accordingly.

### Microbial strains and their growth conditions

Gram-negative bacteria *S. marcescen*s (MTCC No. 2645) used in this study was purchased from the microbial type culture collection (MTCC), Chandigarh, India. Following subculturing on GM3 medium with 2% agar at 37 °C for 24 h, the bacterial strain was propagated in broth cultures for all experimental procedures.

### Determination of minimum inhibitory concentration (MIC) and Minimum Bactericidal concentration (MBC)

The minimum inhibitory concentrations (MICs) of CA and SA against *S. marcescens* were determined by the broth microdilution method with slight modifications. Briefly, a 10⁶ CFU mL^−1^ of bacterial suspension was inoculated into 96-well microtiter plates containing serial dilutions of CA or SA (10–2000 µg mL^−1^). Control wells received 1% (v/v) acetone. Plates were incubated at 37 °C for 24 h at 100 rpm. Bacterial viability was assessed using MTT assay as described by Vishwakarma and Sirisha (2020). The MIC was defined as the lowest concentration of the compound that visibly inhibited bacterial growth (M100 Performance Standards for Antimicrobial Susceptibility Testing 2019; Shaikh et al. 2023; Pandey et al. 2024b).

To determine the minimum bactericidal concentration (MBC), 100 µL from the MIC wells showing no visible growth were plated onto GM3 agar plates and incubated at 37 °C for 24 h. MBC was recorded as the lowest concentration that resulted in no colony formation (M100 Performance Standards for Antimicrobial Susceptibility Testing 2019; Shaikh et al. 2023; Vishwakarma et al. 2022).

### Growth kill assay analysis

The effect of CA and SA on bacterial growth dynamics was assessed using a growth-kill assay. *S. marcescens* cultures (10⁸ CFU mL^−1^) were incubated with varying concentrations of CA and SA (0–MIC) in 96-well microtiter plates. Bacterial growth was monitored by measuring absorbance at 595 nm over a 48 h period. Growth curves were generated by plotting optical density (Y-axis) against time (X-axis) to evaluate the temporal antibacterial effects of both compounds (Pandey et al. 2024b; Vishwakarma and Sirisha 2020).

### Effect of phenolic compounds on the colony forming ability of *S. marcescens*

To assess the impact of phenolic compounds on the colony-forming ability of *S. marcescens*, cultures (10⁶ CFU mL^−1^) were treated with increasing concentrations of CA and SA and incubated at 37 °C, 100 rpm for 24 h. Treated and control cultures were then serially diluted to 10⁸ CFU mL^−1^. From each dilution, 100 µL was spread on GM3 agar plates and incubated at 37 °C for 24 h. Colony formation was assessed post-incubation (Pandey et al. 2024b; Zhou et al. 2012).

### Flow cytometry analysis of the effect of phenolic compounds on *S. marcescens* cell viability

To distinguish live and dead *S. marcescens* cells, a dual-staining protocol was performed using the BD Cell Viability Kit (BD Biosciences). Cultures were treated with varying concentrations (0 to MIC) of CA and SA and incubated at 37 °C, 100 rpm for 24 h. After treatment, 50 µL of each sample was stained with 42 µmol/L thiazole orange (TO) and 4.3 mmol/L propidium iodide (PI) for 20 min in the dark, following the manufacturer's instructions. Unstained samples and isotype-matched controls were used for gating and optimization. Samples were analysed using a BD FACSVerse flow cytometer, acquiring 30,000 events per sample (Michelutti et al. 2020).

### Potential of phenolic compounds to induce apoptosis like cell death in *S. marcescens*

Phosphatidylserine (PS) exposure, an early indicator of apoptosis-like death, was evaluated using the Annexin V–FITC/PI Apoptosis Detection Kit (MCE, USA). *S. marcescens* cells were treated with varying concentrations of CA and SA and incubated at 37 °C at 120 rpm for 1 h. Following treatment, cells were harvested by centrifugation and resuspended in PBS. To each sample, 5 μL of Annexin V–FITC, 100 μL of binding buffer, and 5 μL of PI were added, followed by a 15-min incubation at room temperature in the dark. Subsequently, samples were diluted with 400 μL of Annexin V binding buffer and analysed immediately using a FACSCalibur flow cytometer (BD, Biosciences USA) (Yun and Lee 2016).

### Biofilm inhibition assay

The anti-biofilm efficacy of phenolic acids was assessed using a modified crystal violet (CV) staining method. *S. marcescens* cultures (OD₆₀₀ = 0.3) were treated with varying concentrations (0–MIC) of CA and SA and incubated at 37 °C for 24 h. Following incubation, non-adherent cells were removed by gently washing the wells with sterile distilled water. The attached biofilms were stained with 1% CV for 30 min, then washed three times to remove excess dye. Bound CV was solubilized in absolute ethanol, and biofilm biomass was quantified by measuring absorbance at 595 nm (Bazargani and Rohloff 2016; Martino et al. 2003; Graziano et al. 2015; Sasirekha et al. 2015; Pandey et al. 2022).

### Cell surface hydrophobicity assay (CSH)

The cell surface hydrophobicity of *S. marcescens* after phenolic acid treatment was assessed using a hydrocarbon partitioning method. Bacterial suspension were adjusted to an optical density (OD) of 0.6 and treated with varying concentrations of CA and SA ranging from 0 up to their MICs. The cultures were then incubated at 37 °C, at 120 rpm for 24 h. Untreated cells served as a negative control. After incubation, the bacterial cells were mixed with equal volume of toluene and allowed to stand for 30 min to facilitate phase separation. Subsequently, the optical density of the aqueous phase was measured at 600 nm, and the decrease in absorbance relative to controls was used as a measure of bacterial partitioning into the hydrocarbon phase, thereby reflecting cell surface hydrophobicity (Vishwakarma and Sirisha 2020; Chari et al. 2014; Sorongon et al. 1991).

### Biofilm eradication assay

To evaluate the ability of phenolic compounds to disrupt preformed or mature biofilms of *S. marcescens*, a biofilm eradication assay was conducted. For this experiment, biofilms were established by inoculating bacterial suspension (10⁶ CFU mL^−1^) into wells containing 15 mM hydrogen peroxide and incubating at 37 °C, 120 rpm for 24 h. After incubation, planktonic cells were gently removed, and fresh medium containing varying concentrations (0 to MIC) of CA and SA was added to the wells. The plates were then incubated for an additional 24 h under the same conditions. Hydrogen peroxide-induced biofilms without phenolic treatment served as the positive control. Biofilm biomass was quantified using the crystal violet staining method as described previously. The biofilm-eradicating potential of CA and SA was determined relative to the positive control (Pandey et al. 2024b; Geier et al. 2008).

### Quantification of extracellular polymeric substance (EPS) assay

To evaluate the effect of phenolic compounds on the mature biofilm’s EPS layer, the total EPS content of the biofilms was assessed using this assay. In this experiment, biofilms were preformed as mentioned above and treated with different concentrations of CA and SA separately. These cultures along with appropriate controls were incubated for 24 h at 37 °C, 120 rpm. Post incubation, the cultures were treated with 10% Trichloroacetic Acid (TCA) and an equal volume of acetone, and the cell suspensions were incubated overnight at 4 °C. The samples were then centrifuged at 8000 rpm for 5 min. Total EPS content was quantified by comparing the weight of the sample pellet treated with phenolic compounds with that of the untreated control pellet (Nithya et al. 2014).

### Quantification of eDNA content

To evaluate the impact of CA and SA on extracellular DNA (eDNA) content within the EPS matrix, eDNA was extracted following the protocol by Vishwakarma et al. (2019). Quantification was performed by measuring the absorbance ratio at 260/280 nm, and eDNA yield from phenolic acids-treated samples were compared with those from untreated control cells (Wang et al. 2011).

### DNA binding assay

Extracellular DNA (eDNA) was extracted from *S. marcescens* biofilms as described in the above protocol. For the binding assay, 100 ng of biofilm-derived eDNA was incubated with 10 µM ethidium bromide (EtBr) in a black 96-well microplate at room temperature in the dark for 2 h to allow eDNA–EtBr complex formation. Following incubation, varying concentrations (0 to 2MIC) of CA and SA were added separately to the complexes, and the final reaction volume was adjusted to 200 µL with 10 mM Tris–HCl buffer (pH 7.2). Samples were further incubated for 1 h at room temperature in the dark. Fluorescence was measured using a Tecan spectrofluorometer with excitation at 500 nm and emission recorded over 550–750 nm. A reduction in fluorescence intensity was taken as evidence of disruption of the eDNA–EtBr complex, indicating competitive binding or intercalation interference by the phenolic acids (Raza et al. 2023).

### Scanning electron microscope (SEM) analysis

*S marcescens* cultures were grown on coverslips at 37 °C in GM3 liquid medium for 24 h. The cultures were then treated with 15 mM H_2_O_2_ to form biofilms on these coverslips. These coverslips were then treated with 1/2 MIC and MIC of CA and SA separately for 24 h. The untreated biofilm control was also maintained. Post incubation, the coverslips were washed with PBS thrice and then fixed with 2.5% glutaraldehyde for 12 h. The coverslips were dehydrated the next day using graded ethanol (50% – 100%) and dried in a desiccator for 24 h. The dried biofilms (control and treated) were coated with platinum and observed using a scanning electron microscope (FEI Quanta 200 (XT Microscope Control) set to magnification of 20,000 X (Vishwakarma et al. 2022; Yan et al. 2017).

### Confocal laser scanning microscopy (CLSM) investigation of the impact of phenolic acids on biofilms of *S. marcescens*

Biofilms of *S. marcescens* were grown on sterile coverslips and treated with 1/2 MIC and MIC of CA and SA, incubated for 24 h at 37 °C, and then gently washed twice with sterile Milli Q water to remove any unattached and planktonic cells. Biofilms (control and treated) were then stained with two different fluorophores to visualise distinct extracellular matrix components.

Exopolysaccharide residues inside the biofilm matrix were stained with TRITC-conjugated Concanavalin A (Con A) (Sigma, USA) at 1 mg mL^−1^ in sterile milliQ water. These residues were evident in the green channel. The eDNA component was stained with propidium iodide (PI) (Sigma, USA) at a 50 μg mL^−1^ in sterile milli-Q water, detected in red channel. Following a series of treatments with 500 μL of each staining solution, the coverslips were rinsed and incubated at room temperature in dark for 20 min. After incubation, the coverslips were rinsed with milli-Q water thoroughly, and gently blotted dry on tissue paper for two min. These coverslips were placed on sterile slides using mounting solution, and a Zeiss LSM 980 with 63 X immersion lenses was used for confocal laser scanning microscopy (CLSM). At  488 nm, an argon laser was used to excite ConA fluorophores, and at  560 nm, a helium–neon laser was used to stimulate PI fluorophores. ConA and PI emission filters were set to 493- 561 and 565-727  nm, respectively. A Zeiss LSM 910 with a 63X/1.40 oil immersion lens and hybrid detectors was used to capture the images. Z-stacks were taken at random points along the biofilm thickness at 0.125 μm intervals, stored as CNZ files, and then analysed with Zeiss Zen software (Gokhale et al. 2024; Di Somma et al. 2020; Heydorn et al. 2000). For quantitative analysis, the fluorescence areas (measured in square pixels) were calculated from images of three independent biofilm samples, each collected from two different locations..

## Quantification of QS-induced virulence factors

### Lipase assay

Control and phenolic compounds treated *S. marcescens* cells were centrifuged at 10,000 × *g* for 10 min at 4 °C. The resulting pellets were resuspended in TE buffer (pH 8), and cells were lysed by using sonication. Lysates were centrifuged at 9500 rpm for 10 min at 4 °C, and the resulting cell free supernatant was collected and used for lipase activity. The lipase activity of the supernatant was measured using P-nitrophenyl palmitate (pNPP) as the substrate. The substrate solution was prepared by mixing solutions A (3 mg of pNPP in 1 mL isopropanol) with solution B (10 mg of gum arabic and 40 mg of Triton-X in 9 mL of 50 mM Tris–HCl buffer pH 8). The substrate mixture was supplemented with 0.1 mL of the cell-free supernatant, incubated for 20 min at 60 °C in a water bath. Lipase activity was quantified by measuring the absorbance at 410 nm (Eko Sukohidayat et al. 2018).

### Prodigiosin pigment assay

A 0.5 OD culture of *S marcescens* was treated with different concentrations of CA and SA (0-MIC) and incubated at 37 °C for 24 h. The cells were then centrifuged at 10,000 rpm for 10 min at 25 °C. The resulting pellet was resuspended in 4% 1N HCL prepared in ethanol. This mixture was vortexed and incubated for 15 min at room temperature to extract the pigment. Prodigiosin content was then quantified by measuring the absorbance at 534 nm (Khadar et al. 2012).

### Protease assay

For this assay, a suspension of *S. marcescens* (10^6^ CFU mL^−1^) was treated with varying concentrations of CA and SA and incubated at 37 °C for 24 h. The cells were then centrifuged at 11,000 rpm for 20 min at 4 °C. To the supernatant an equal amount of azocasein solution was added, incubated for 2 h at 37 °C in shaking conditions. The reaction was terminated by adding an equal amount of 10% TCA, incubated at -20 °C for 20 min. The mixture was then centrifuged at 12,000 rpm for 15 min at 25 °C. To the final supernatant, half volume of 1 M NaOH was added and the absorbance was measured at 440 nm (Salini and Pandian 2015).

### Urease assay

To assess urease activity in control and treated cells, *S. marcescens* cultures (0.3 OD) were treated with varying doses of CA and SA (0-MIC) and incubated at 37 °C for 24 h. After incubation, the supernatant was extracted by centrifuging at 8499 × *g* for 5 min at 25 °C. For the assay, 0.5 mL of substrate (2% urea) was mixed with 0.1 mL of bacterial cell supernatant and incubated for 3 h at 37 °C in a water bath. Post incubation, 0.1 mL of Nessler's reagent was added, mixed well, and allowed to sit at room temperature for 5 min. Urease production was then measured at 530 nm using spectrophotometer (Kauffmann and Møller 1995).

### QS induced swimming and swarming motility assay

For swimming and swarming motility assays, GM3 agar media plates were prepared with 0.3% and 0.5% agar respectively. Then, varying amounts of CA and SA were added to these plates and allowed the plates to solidify. Control plates were prepared in the same manner without phenolic acids. A10 μL aliquot of *S. marcescens* suspension (10^6^ CFU mL^−1^) was inoculated at the centre of solidified agar plates, which were then incubated for 48 h at 37 °C (Kearns 2010).

## Results

### Antibacterial activity of CA and SA against *S. marcescens:*

#### Determination of minimum inhibitory and bactericidal concentrations (MIC and MBC)

The minimum inhibitory concentrations of CA and SA to inhibit the visible growth of the respiratory tract infection organism *S. marcescens* were 700 µg mL^−1^ and 1000 µg mL^−1^ respectively. These findings highlight the greater antibacterial potency of coumaric acid relative to syringic acid. To further delineate the mode of action, MBC assay was performed. CA demonstrated a bactericidal effect, effectively killing *S. marcescens* cells, while SA exhibited a bacteriostatic effect, suppressing bacterial proliferation without inducing complete cell death. Taken together, these results demonstrate that CA exhibits stronger antibacterial activity than SA, against *S. marcescens* infections.

### Growth kill assay and colony forming ability assay

**Fig. 1:**
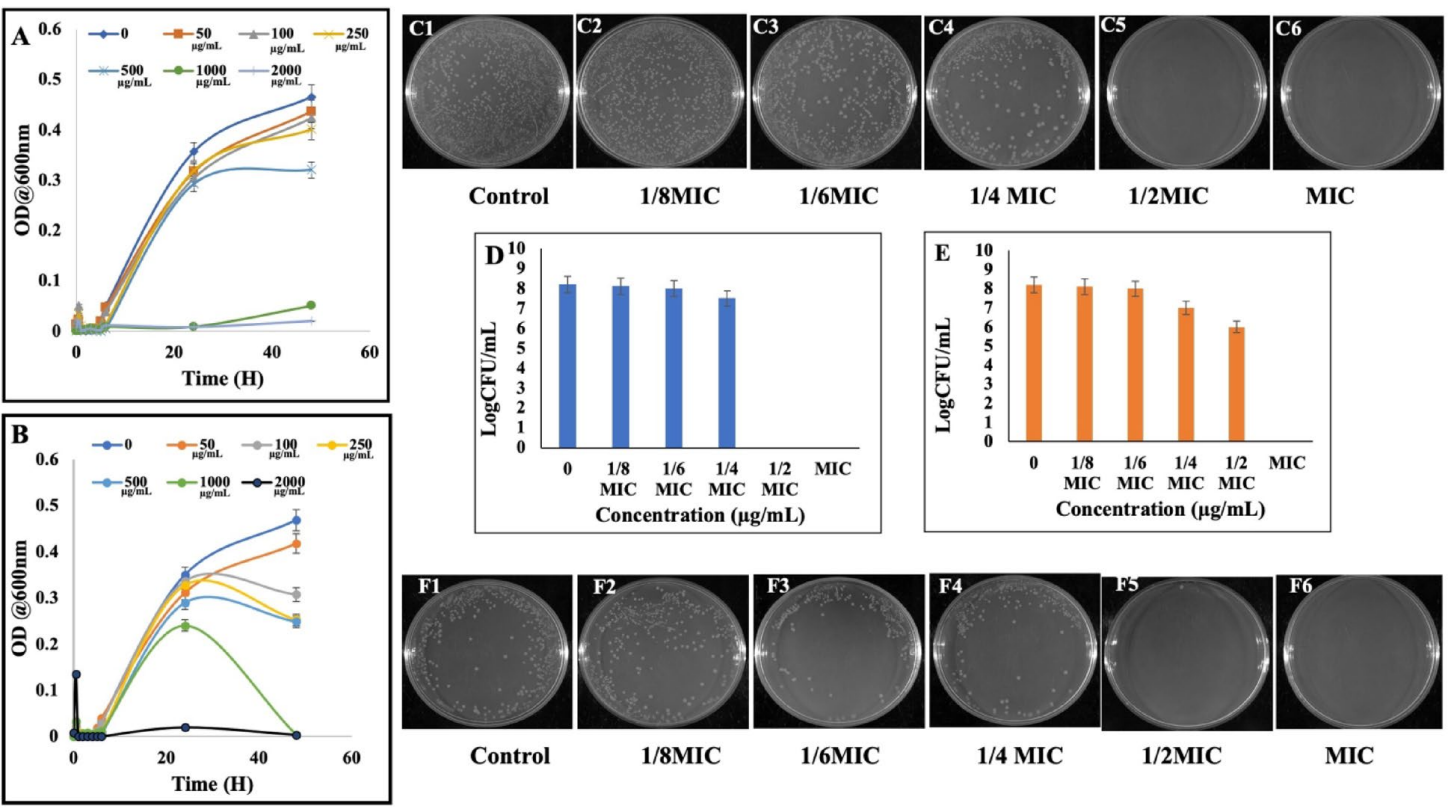
Growth-kill kinetics of *S. marcescens* treated with (**A**) coumaric acid and (**B**) syringic acid over a 24 h period, showing concentration-dependent inhibition. Colony-forming ability assays (C1–C6 for coumaric acid; D1–D6 for syringic acid) show a concentration-dependent decrease in viable colonies, indicating the bactericidal potential of both compounds. Values represent the mean of three independent experiments; statistical significance was considered at *P* < 0.05

The absorbance-time growth curves revealed a strong positive correlation between bacterial growth inhibition over time with increased concentrations of CA and SA. Initially, all concentrations exhibited similar growth patterns; however, the number of viable cells gradually decreased with extended time of incubation. In the case of CA, complete growth inhibition was observed at ~ 700 µg mL^−1^ after 24 h, whereas SA achieved comparable inhibition only at 1000 µg mL^−1^ (Fig. 1A, B).

The colony-forming ability assay further demonstrated a marked reduction in *S. marcescens* colony growth after 24 h of treatment with phenolic acids compared to the untreated control. Both CA and SA completely inhibited colony formation from 1/2MIC and MIC (Fig. 1C, F).

### Flow cytometry analysis of cell viability post phenolic acids treatment in *S. marcescens*

**Fig. 2:**
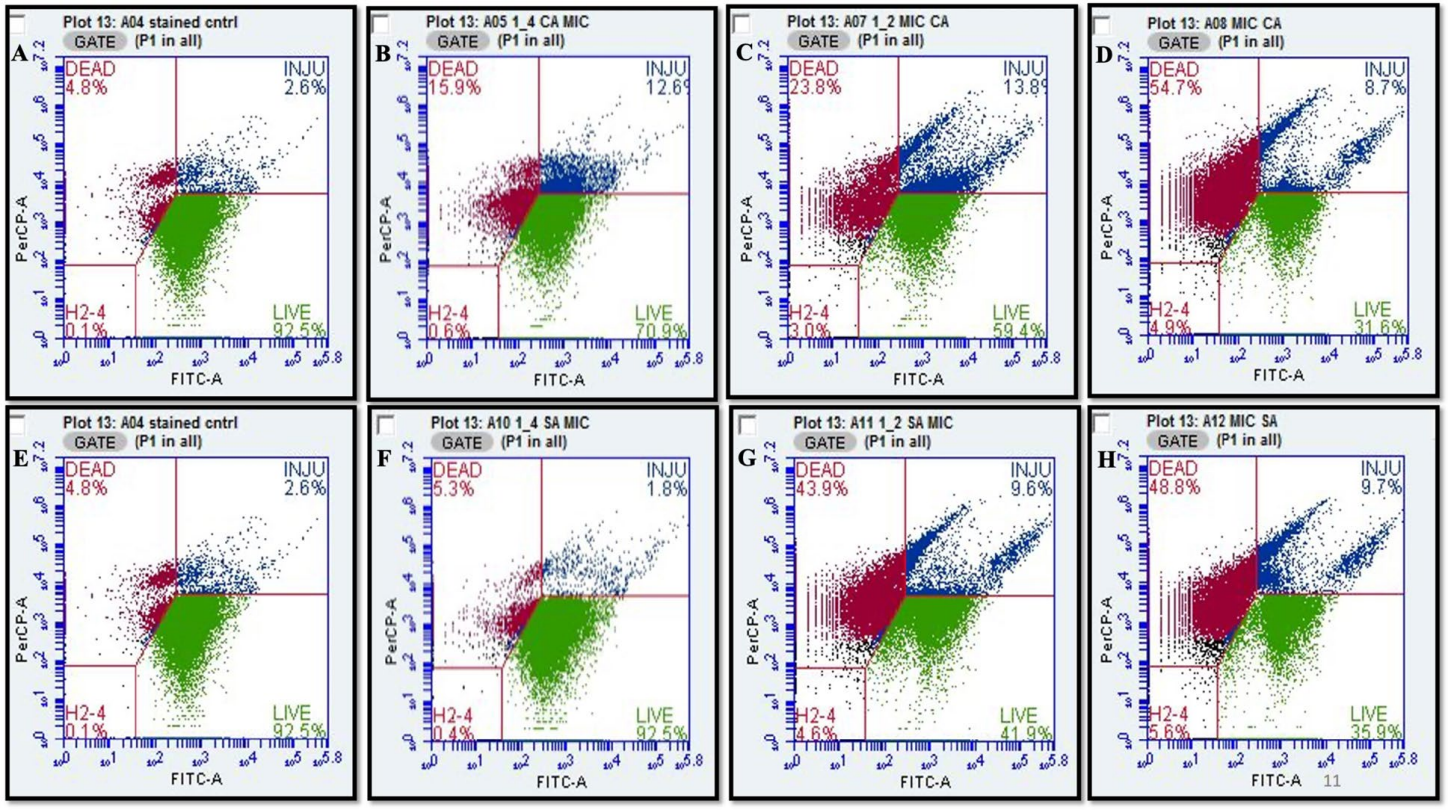
Flow cytometry dot plots illustrating the viability of *S. marcescens* cells with varying concentrations of coumaric acid (CA) and syringic acid (SA): (**A**) untreated control for CA, (**B**) 1/4MIC CA, (**C**) 1/2MIC CA, (**D**) MIC CA, (**E**) untreated control for SA, (**F**) 1/4 MIC SA, (**G**) 1/2 MIC SA, and (**H**) MIC SA. Data represent the mean of three independent experiments

To gain mechanistic insights into the antibacterial activity of these phenolic acids, a cell viability assay was performed using flow cytometry. *S. marcescens* cells exposed to varying concentrations of CA and SA were analysed with the BD Cell Viability Kit. Flow cytometric analysis revealed that CA induced approximately 55% cell death and 9% cellular injury (Fig. 2A-D), whereas SA caused 49% cell death and 10% injury in a concentration-dependent manner (Fig. 2E-H). These findings highlight the capacity of phenolic acids to compromise bacterial viability, inducing significant levels of cell death and sub-lethal injury in *S. marcescens*.

### Apoptosis like cell death

**Fig. 3:**
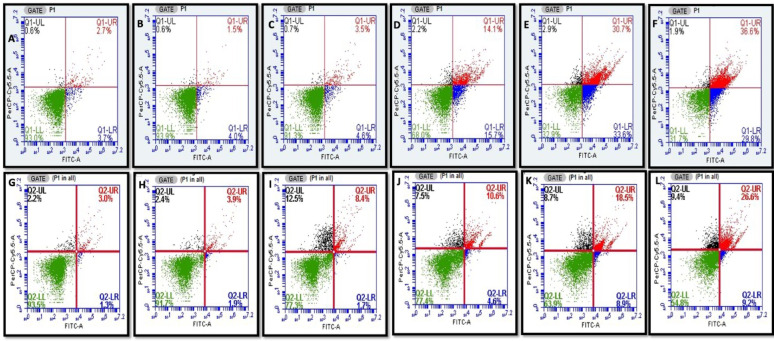
Annexin V staining assay detecting phosphatidylserine externalization in *S. marcescens* cells treated with phenolic compounds, indicating apoptosis-like cell death. panels show (**A**) untreated control for coumaric acid (CA), (**B**) 1/8 MIC CA, (**C**) 1/4 MIC CA, (**D**) 1/2 MIC CA, (**E**) MIC CA, (**F**) 2 × MIC CA, (**G**) untreated control for syringic acid (SA), (**H**) 1/8 MIC SA, (**I**) 1/4 MIC SA, (**J**) 1/2 MIC SA, (**K**) MIC SA, and (L) 2 × MIC SA. Data represent the mean of three independent experiments

Phosphatidylserine (PS), normally localised to the inner membrane leaflet, translocates to the outer leaflet during apoptosis-like cell death. This translocation was detected using Annexin V-FITC, which selectively binds to exposed PS. Flow cytometry with Annexin V-FITC/PI dual staining enabled differentiation of cell death stages: Annexin V-FITC⁺/PI⁻ (early apoptosis), Annexin V-FITC⁺/PI⁺ (late apoptosis), and Annexin V-FITC⁻/PI⁺ (necrosis). Following treatment with CA, *S. marcescens* showed 29.8% early apoptotic, 36.6% late apoptotic, and 1.9% necrotic cells (Fig. 3A-F). Treatment with SA resulted in 9.2% early apoptotic, 26.6% late apoptotic, and 9.4% necrotic cells (Fig. 3G-L), demonstrating that both compounds induce apoptosis-like cell death, albeit with varying efficacy profiles.

### Antibiofilm potential of phenolic acids in *S. marcescens*

#### Biofilm inhibition assay and cell surface hydrophobicity assay

**Fig. 4:**
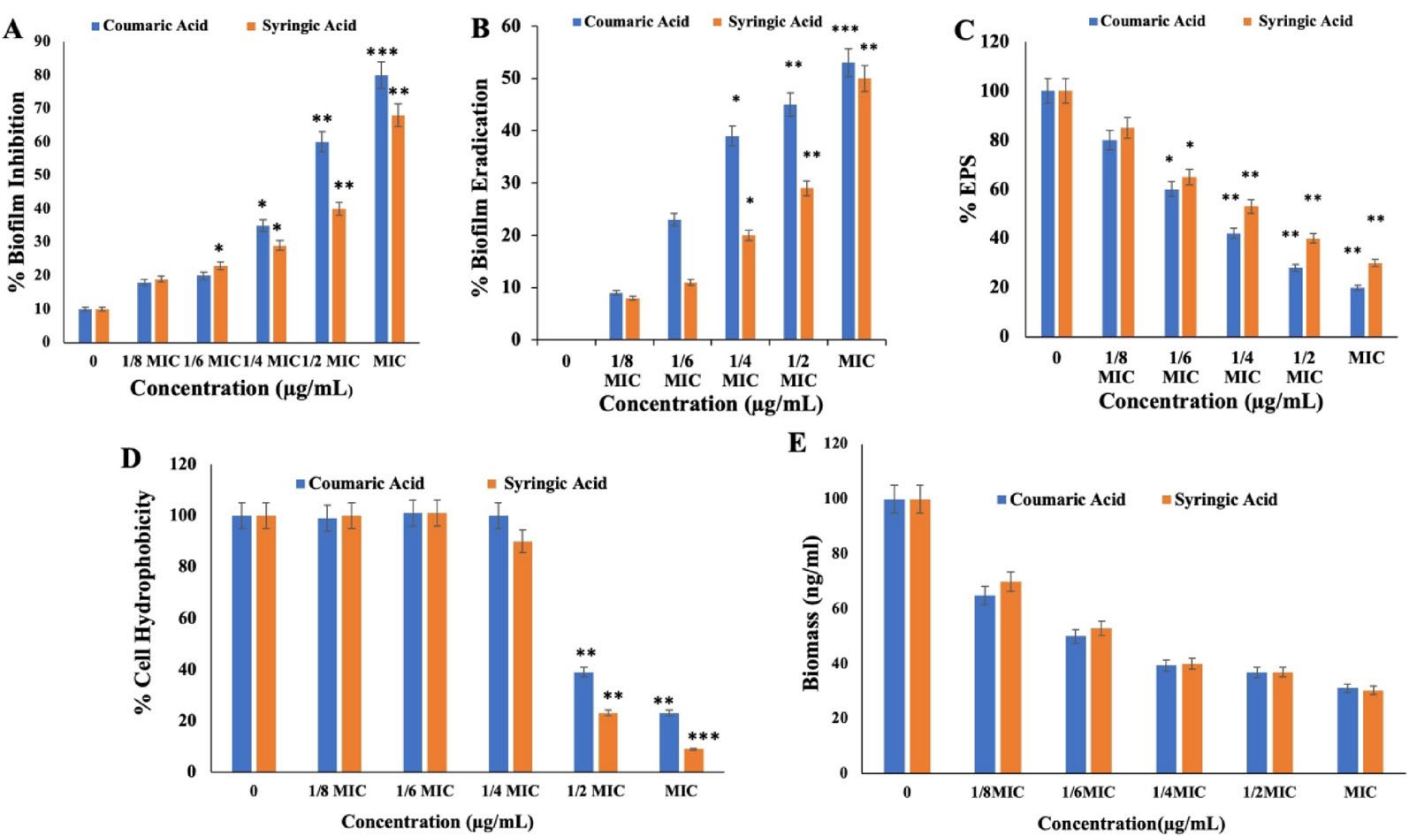
Antibiofilm activity of phenolic compounds against *S. marcescens:* (**A**) inhibition of biofilm formation, (**B**) disruption of established biofilms, (**C**) quantification of extracellular polymeric substances (EPS), (**D**) measurement of cell surface hydrophobicity, and (**E**) extracellular DNA (eDNA) quantification. Results represent the mean ± SD of three independent experiments. *P* < *0.05*

The antibiofilm potential of phenolic acids was evaluated using a standard biofilm inhibition assay. A marked, concentration-dependent reduction in biofilm formation was observed upon treatment with CA and SA. At MIC, CA inhibited biofilm formation to a maximum of 80%, while SA achieved ~ 70% inhibition, indicating their strong potential to disrupt initial biofilm development. (Fig. 4A). Cell surface hydrophobicity assay was performed to assess the impact of phenolic acids on the hydrophobicity of the bacterial cell membrane. *S. marcescens* treated with varying concentrations of CA exhibited upto 80% reduction in cell surface hydrophobicity, while SA caused a 90% decrease compared to the untreated controls (Fig. 4D). These results suggest that the phenolic acids significantly alter bacterial cell surface hydrophobicity, a critical factor for initial bacterial adhesion, thereby inhibiting biofilm formation.

#### Biofilm eradication assay and quantification of EPS

A biofilm eradication assay was conducted to evaluate the effectiveness of phenolic acids in eliminating pre-formed biofilms. Biofilms of *S. marcescens* were established under oxidative stress conditions, then treated with varying concentrations of the CA and SA separately. Treatment with CA disrupted approximately 50% of the matured preformed biofilms at its MIC, while SA achieved a ~ 47% disruption at its MIC (Fig. 4B).

The EPS quantification assay was utilized to evaluate the ability of phenolic acids to disrupt the protective matrix of mature bacterial biofilms. Increasing concentrations of CA and SA resulted in ~ eight fold reduction in EPS production in *S. marcescens* compared to the untreated controls (Fig. 4C). This demonstrates that both CA and SA efficiently compromise the biofilm’s protective matrix, resulting in the destabilization of mature biofilms and enhancing their susceptibility to clearance.

### Quantification of eDNA and DNA binding assay

In this study, extracellular DNA (eDNA) was quantified from preformed *S. marcescens* biofilms treated with CA and SA, as well as from untreated controls. A significant reduction in eDNA content was observed in treated samples compared to controls. Treatment with MIC of CA and SA resulted in a 0.65-fold decrease in eDNA content within mature biofilms compared to untreated controls (Fig. 4E).

**Fig. 5:**
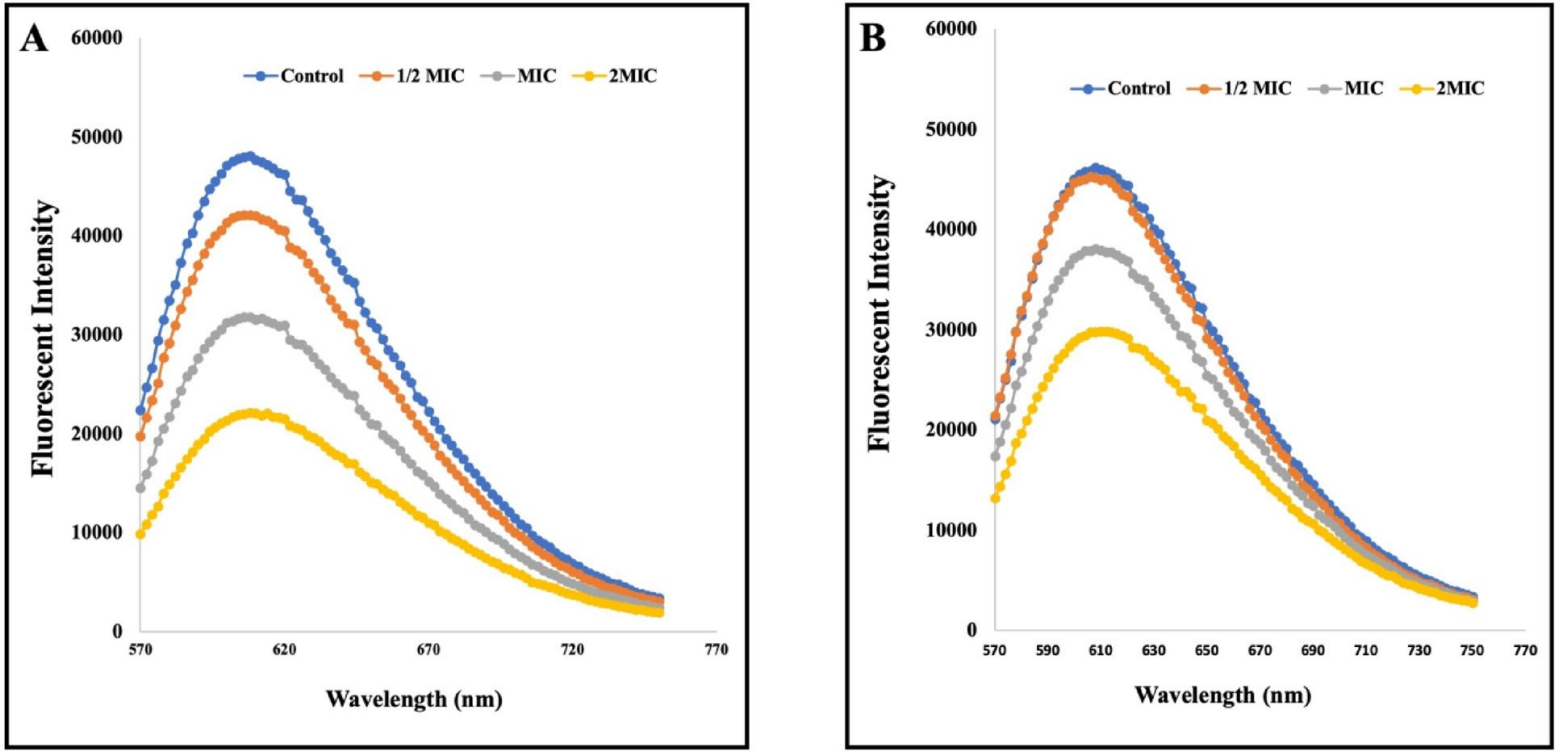
Fluorescence quenching curves of ethidium bromide (EtBr)-DNA complexes in the presence of phenolic acids: (**A**) Competitive displacement of EtBr by coumaric acid, evidenced by a concentration-dependent decrease in fluorescence intensity. (**B**) Competitive displacement of EtBr by syringic acid, showing a similar reduction in fluorescence, indicating binding affinity to eDNA. Data represent the mean of three independent experiments

To further investigate the mechanism by which these phenolic acids target the eDNA component of the EPS matrix, a DNA binding assay was performed using ethidium bromide (EB), a fluorescent intercalator. Competitive fluorometric binding assay revealed a concentration-dependent decrease in EB fluorescence upon addition of CA or SA, indicating that these compounds competitively displaced EB by binding to eDNA (Fig. 5A, B). These findings suggest that CA and SA effectively interact with eDNA, compromising the structural integrity of the EPS matrix and thereby disrupting mature *S. marcescens* biofilms.

### SEM analysis

**Fig. 6:**
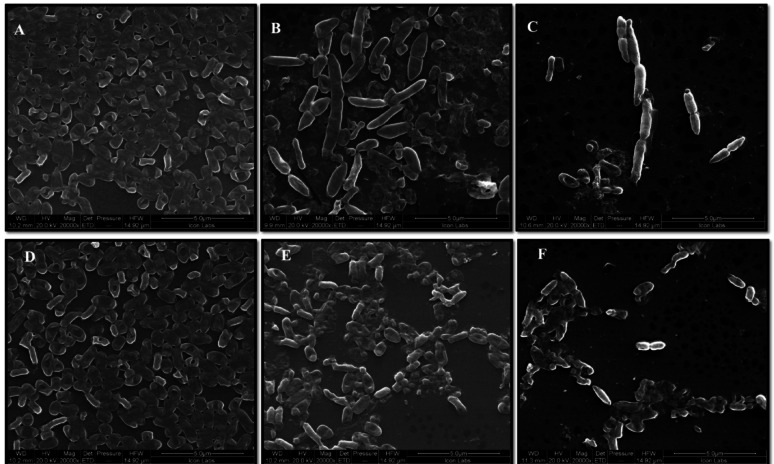
Scanning electron micrographs of *S. marcescens* biofilms: (**A**) Untreated control; (**B**) Treated with 1/2MIC of coumaric acid; (**C**) Treated with MIC of coumaric acid; (**D**) Untreated control for syringic acid; (**E**) Treated with 1/2MIC of syringic acid; (**F**) Treated with MIC of syringic acid. All images captured at 20,000 X magnification; scale bars = 5 μm

Scanning electron microscopy (SEM) analysis demonstrated significant disruption of *S. marcescens* biofilms following treatment with phenolic acids. Untreated controls displayed dense, well-structured biofilm architecture (Fig. 6A, D), whereas biofilms treated with CA and SA showed a substantial decrease in bacterial adhesion and extensive structural disintegration (Fig. 6B, E). Notably, SEM images revealed distinct morphological alterations: CA-treated cells exhibited elongation at MIC levels (Fig. 6C), indicative of cellular stress, while SA-treated samples predominantly consisted of dispersed planktonic cells, reflecting effective biofilm dispersal (Fig. 6F). These marked changes highlight the potent antibiofilm activity of both compounds, emphasizing the capability of these agents to dismantle mature biofilms and inhibit the proliferation of bacteria responsible for respiratory infections.

### Confocal laser scanning microscopy

**Fig. 7:**
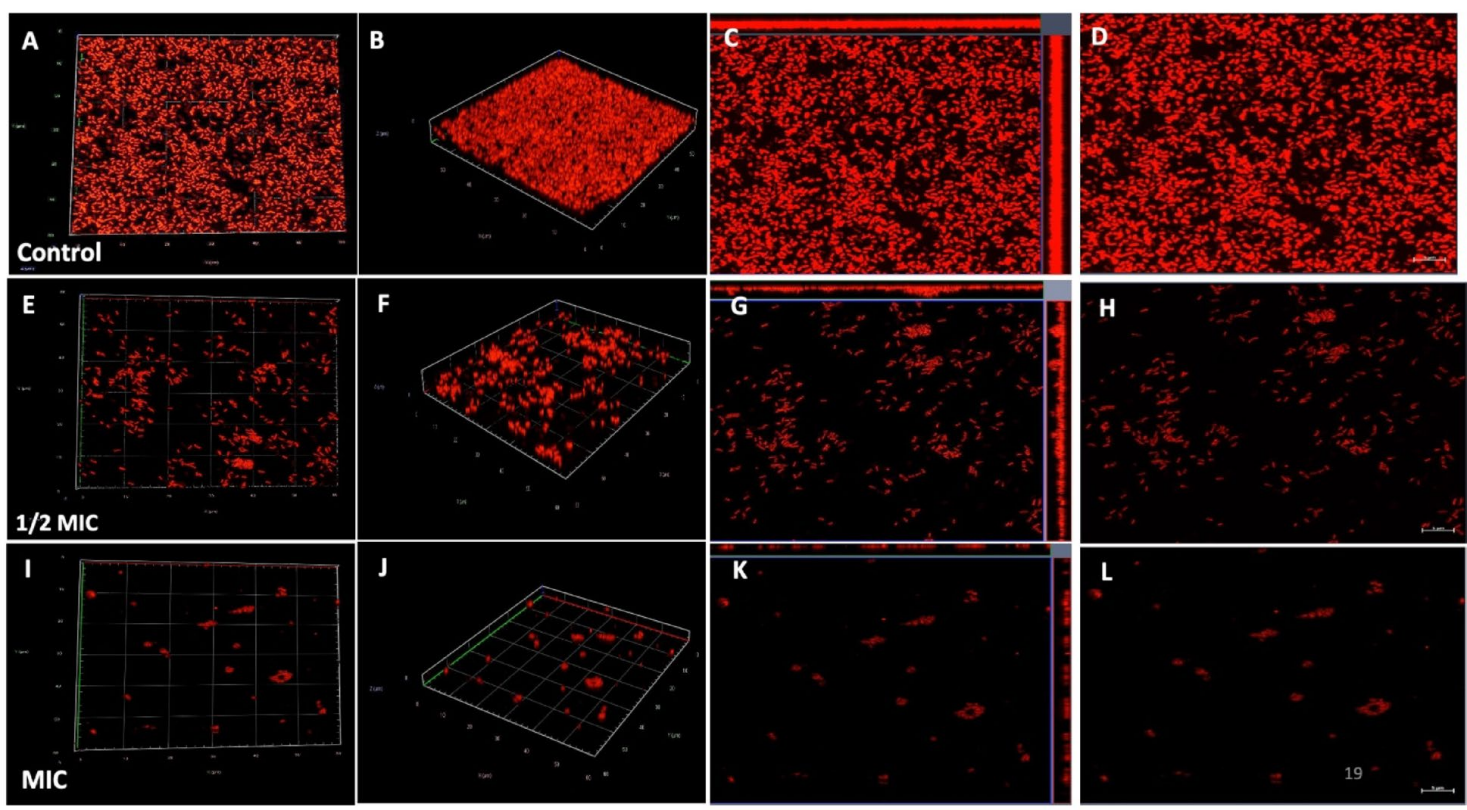
Confocal laser scanning microscopy illustrating the impact of coumaric acid on *S. marcescens* biofilms. (**A**–**D**) Planar and 3D views of untreated control biofilms; (**E**–**H**) Biofilms treated with 1/2MIC of coumaric acid, showing reduced matrix density and partial cell dispersion; (**I**–**L**) Biofilms treated with MIC of coumaric acid, revealing extensive structural disruption, reduced biomass, and predominance of planktonic cells

**Fig. 8:**
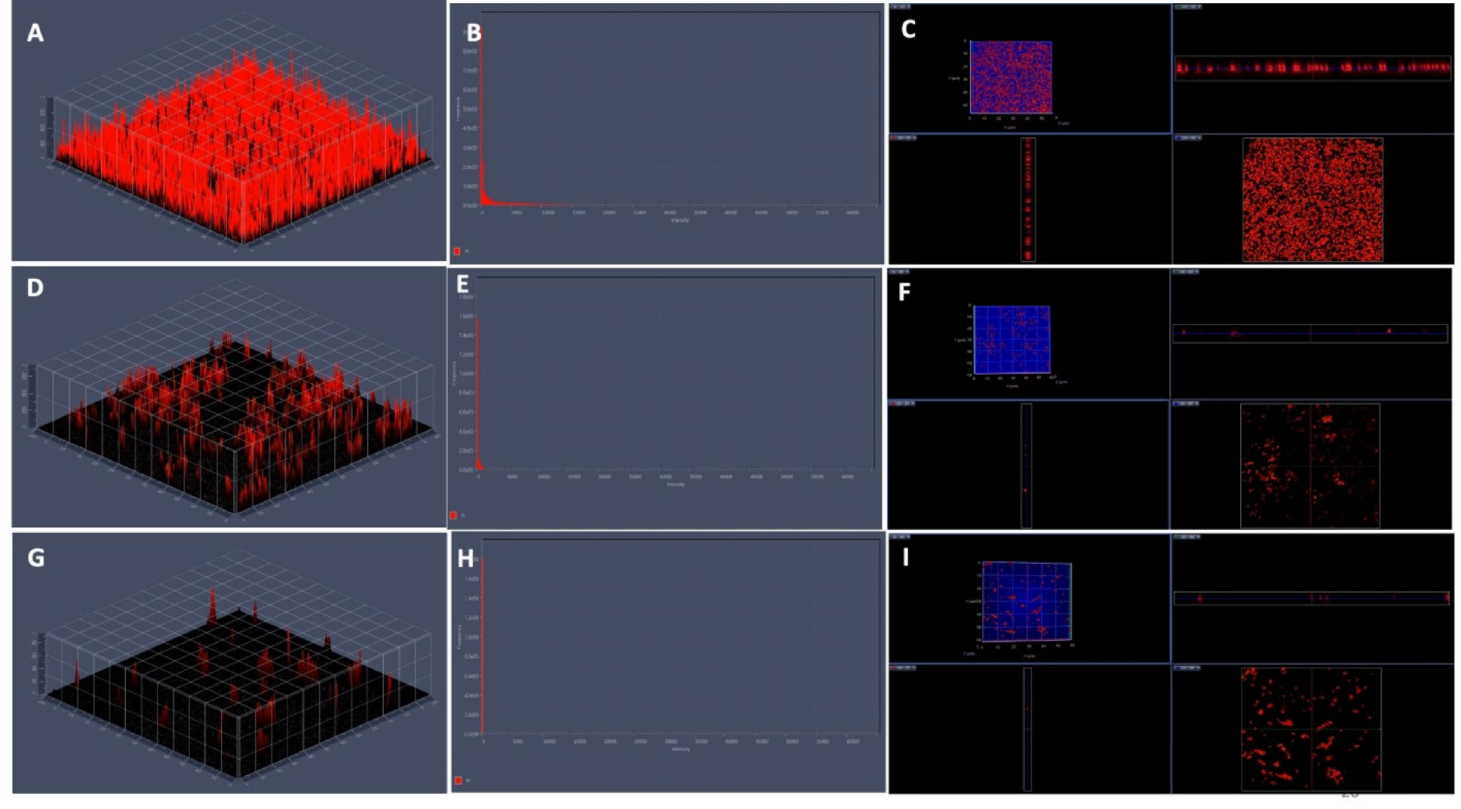
CLSM 3D, 2.5D, and fluorescence intensity images illustrating the impact of coumaric acid on *S. marcescens* biofilms. (**A**–**C**) Control biofilms showing intact structure and high fluorescence intensity upon PI staining. (**D**–**F**) Biofilms treated with 1/2MIC of coumaric acid exhibit disrupted architecture and reduced fluorescence, indicating compromised viability. (**G**–**I**) MIC-CA treated biofilms show substantial structural distortion, diminished matrix, and markedly decreased PI fluorescence, confirming potent antibiofilm activity. Data represent three independent experiments

**Fig. 9:**
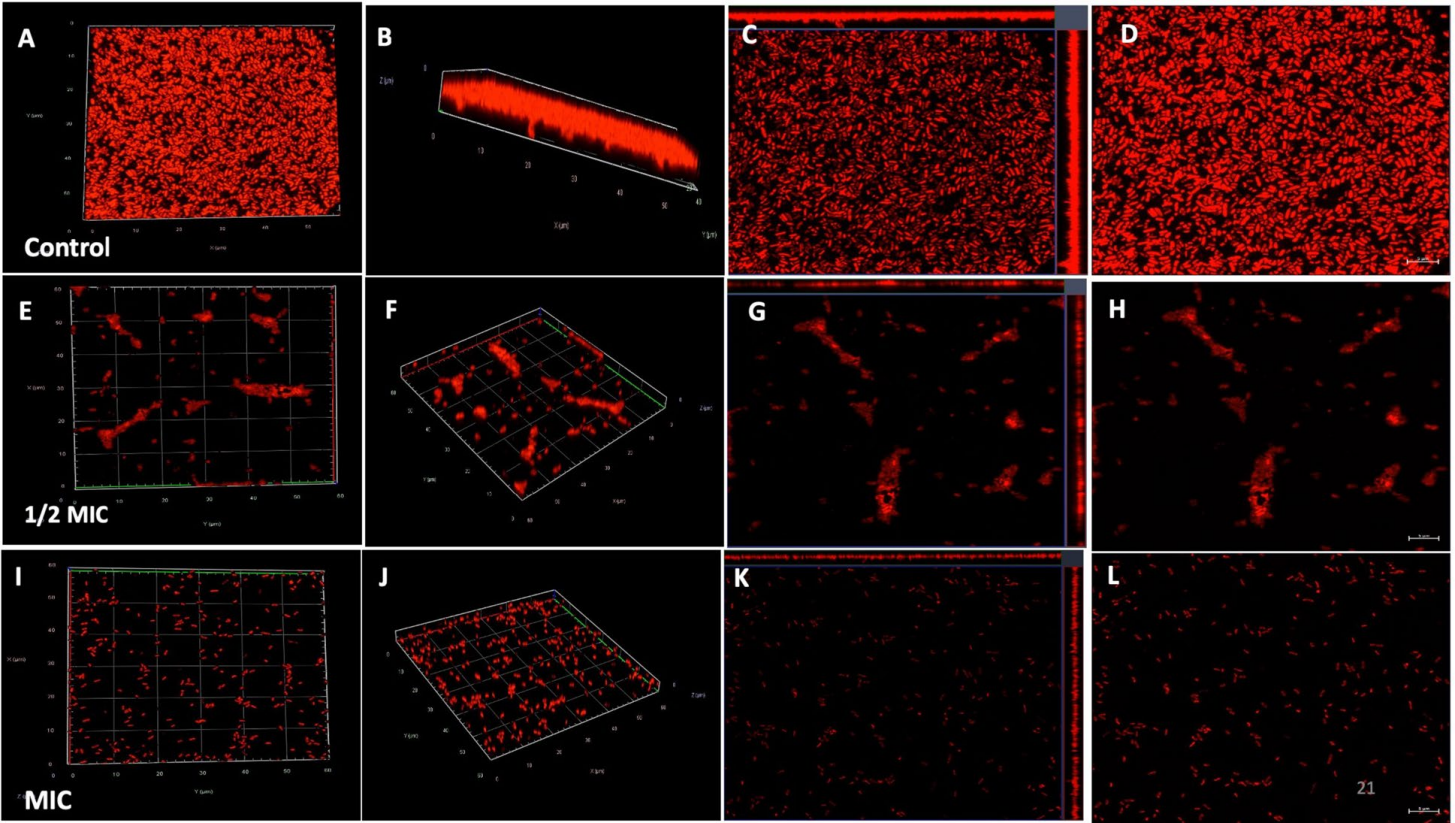
Confocal laser scanning microscopy depicting the effect of syringic acid on *S. marcescens* biofilms. (**A**–**D**) Untreated control biofilms show intact, dense architecture. (**E**–**H**) 1/2MIC treatment leads to disrupted biofilm structure and partial cell release. (**I**–**L**) MIC treatment causes pronounced biofilm degradation, with scattered planktonic cells and minimal residual matrix

**Fig. 10:**
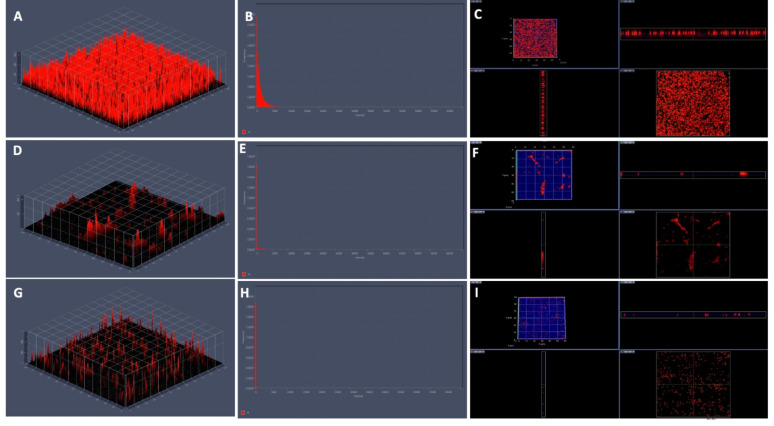
CLSM 3D, 2.5D, and fluorescence intensity images demonstrating the effect of syringic acid on *S. marcescens* biofilms. (**A**–**C**) Control biofilms display intact architecture and strong PI fluorescence. (**D**–**F**) 1/2MIC-SA treatment results in structural disruption and reduced fluorescence. (**G**–**I**) MIC-SA treatment causes pronounced biofilm collapse and minimal PI signal, indicating significant antibiofilm activity. Data represent three independent experiments

Confocal laser scanning microscopy (CLSM) analysis was performed to assess the impact of phenolic acids on biofilm disruption, complementing the SEM findings. Untreated *S. marcescens* biofilms exhibited numerous dense, flake-like structures. Treatment with CA and SA significantly reduced biofilm biomass, which appeared looser and more dispersed with increasing concentrations. Biofilms treated at 1/2 MIC and MIC of both phenolic acids showed markedly lower cell density, appear looser, bubble-like, and less compact architecture compared to controls. These observations indicate that phenolic acids effectively target biofilm matrix components, disrupting mature *S. marcescens* biofilms (Figs. 7, 8, 9 and 10).

**Fig. 11:**
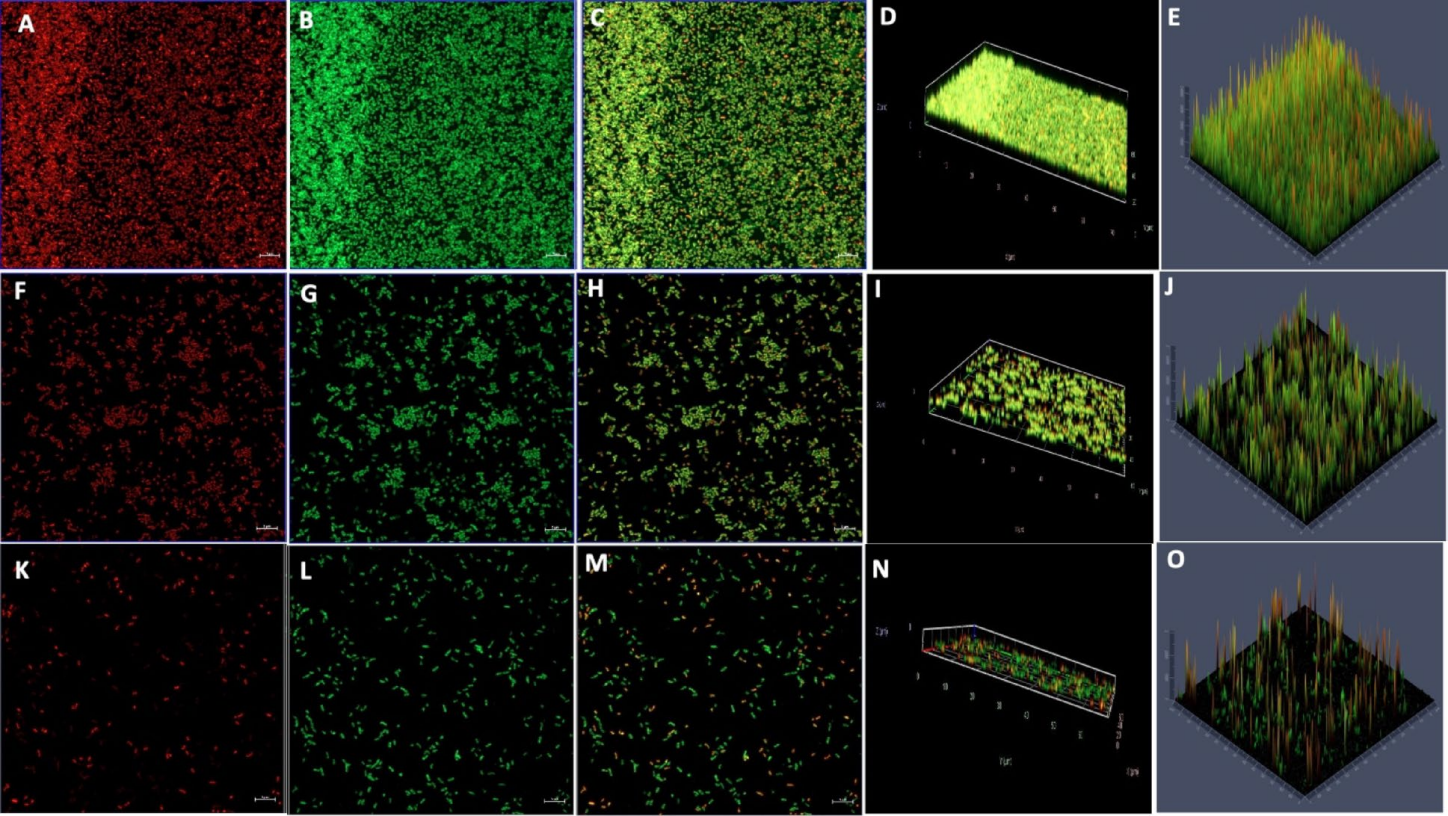
Confocal microscopy of *S. marcescens* biofilms stained with Propidium Iodide (eDNA) and Concanavalin A (EPS) after coumaric acid treatment. (**A**–**E**) Untreated control biofilms showing intact eDNA and EPS matrix. (**F**–**J**) 1/2MIC coumaric acid-treated biofilms with visibly reduced eDNA and EPS signals. (**K**–**O**) MIC-treated biofilms displaying pronounced matrix disruption and weakened fluorescence, indicating degradation of biofilm components

**Fig. 12:**
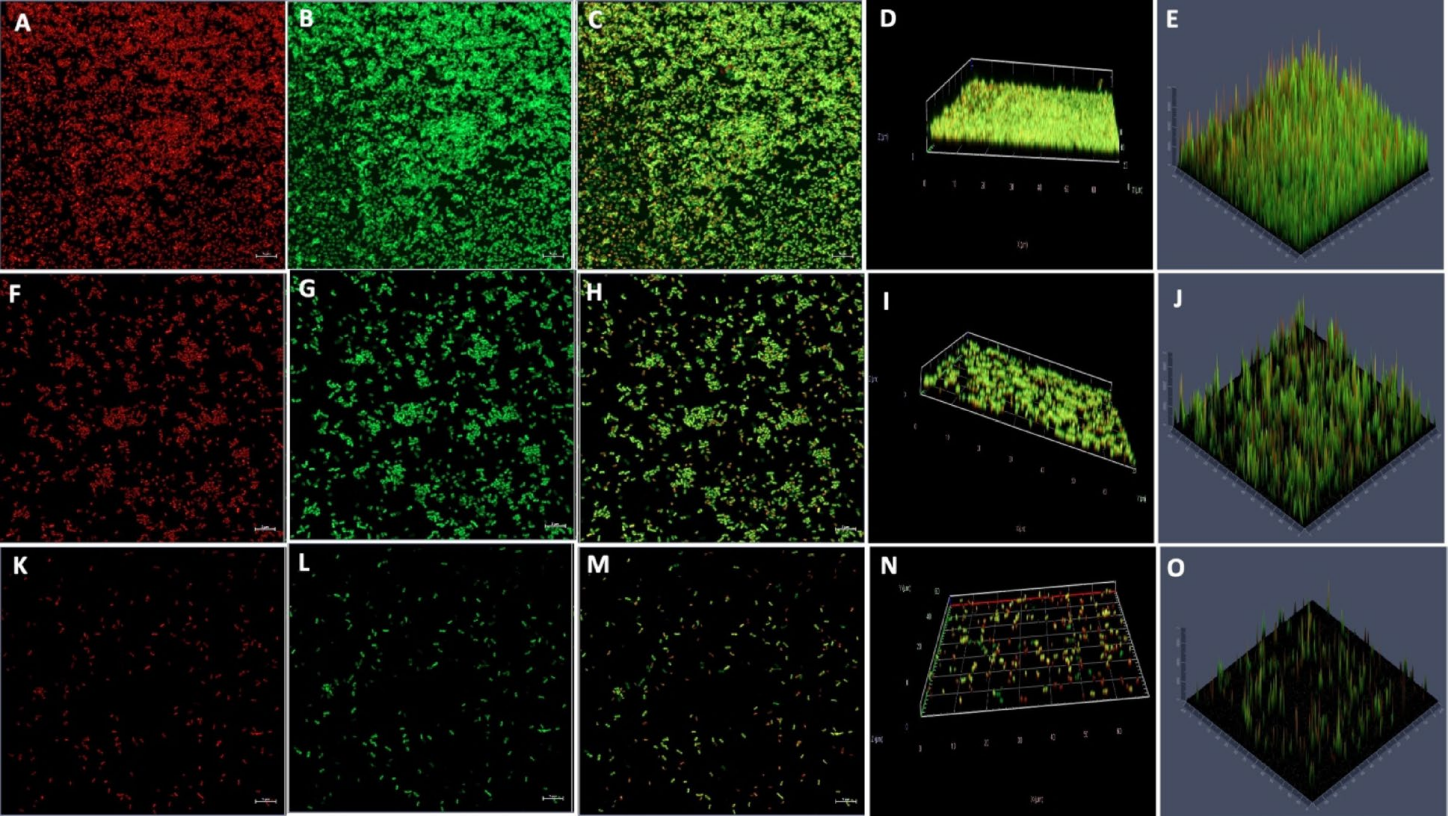
CLSM analysis of *S. marcescens* biofilms stained with Propidium Iodide (eDNA) and Concanavalin A (EPS) following syringic acid treatment. (**A**–**E**) Control biofilms showing intact matrix architecture. (**F**–**J**) 1/2MIC-treated biofilms exhibiting reduced eDNA and EPS fluorescence. (**K**–**O**) MIC-treated biofilms displaying marked structural disruption and significant matrix degradation

Given that the biofilm matrix consists of proteins, eDNA, RNA, and polysaccharides, additional analysis are performed using glass coverslips-grown biofilms to specifically evaluate the inhibitory effects of CA and SA on eDNA and polysaccharide components. Post-treatment staining was performed using Concanavalin A (ConA) to detect extracellular polysaccharides and propidium iodide (PI) for eDNA visualization. The results showed that the mature biofilm matrix in the control samples had a higher polysaccharide content than the treated samples, as indicated by the significantly stronger green fluorescence intensity of ConA-stained *S. marcescens* biofilms. Notably, a marked reduction in green fluorescence was observed as the concentrations of CA and SA increased from 1/2MIC to MIC. This decline in fluorescence suggests that both phenolic acids disrupted the biofilm matrix by reducing its polysaccharide content (Figs. 11 and 12).

**Fig. 13:**
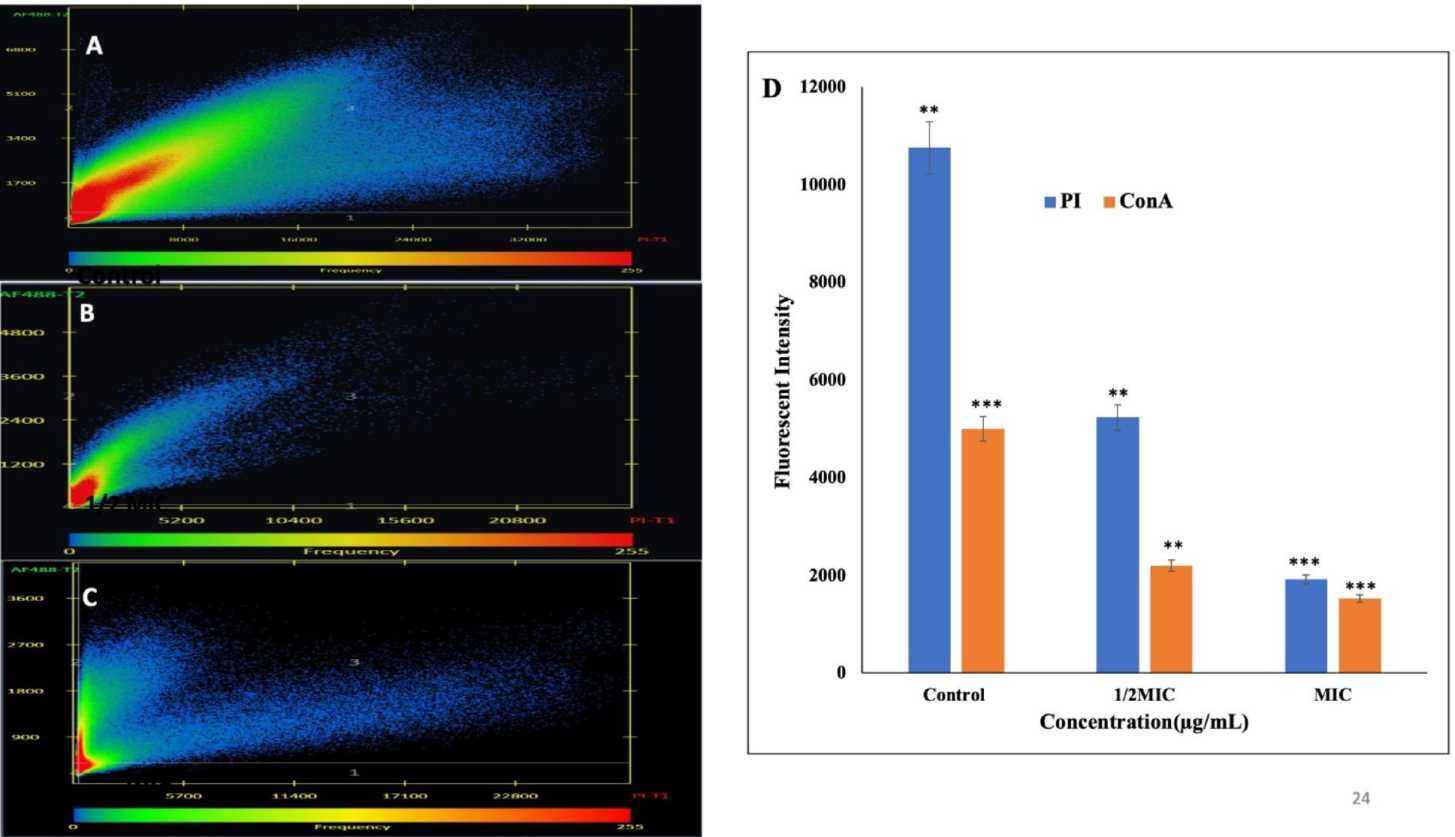
CLSM fluorescence intensity graph of (**A**) Control (**B**) 1/2 MIC-coumaric acid-treated biofilms (**C**) MIC-coumaric acid-treated biofilms. (**D**) The relative fluorescence intensity of matrix components of control and coumaric acid-treated samples within *S. marcescens* biofilms

**Fig. 14:**
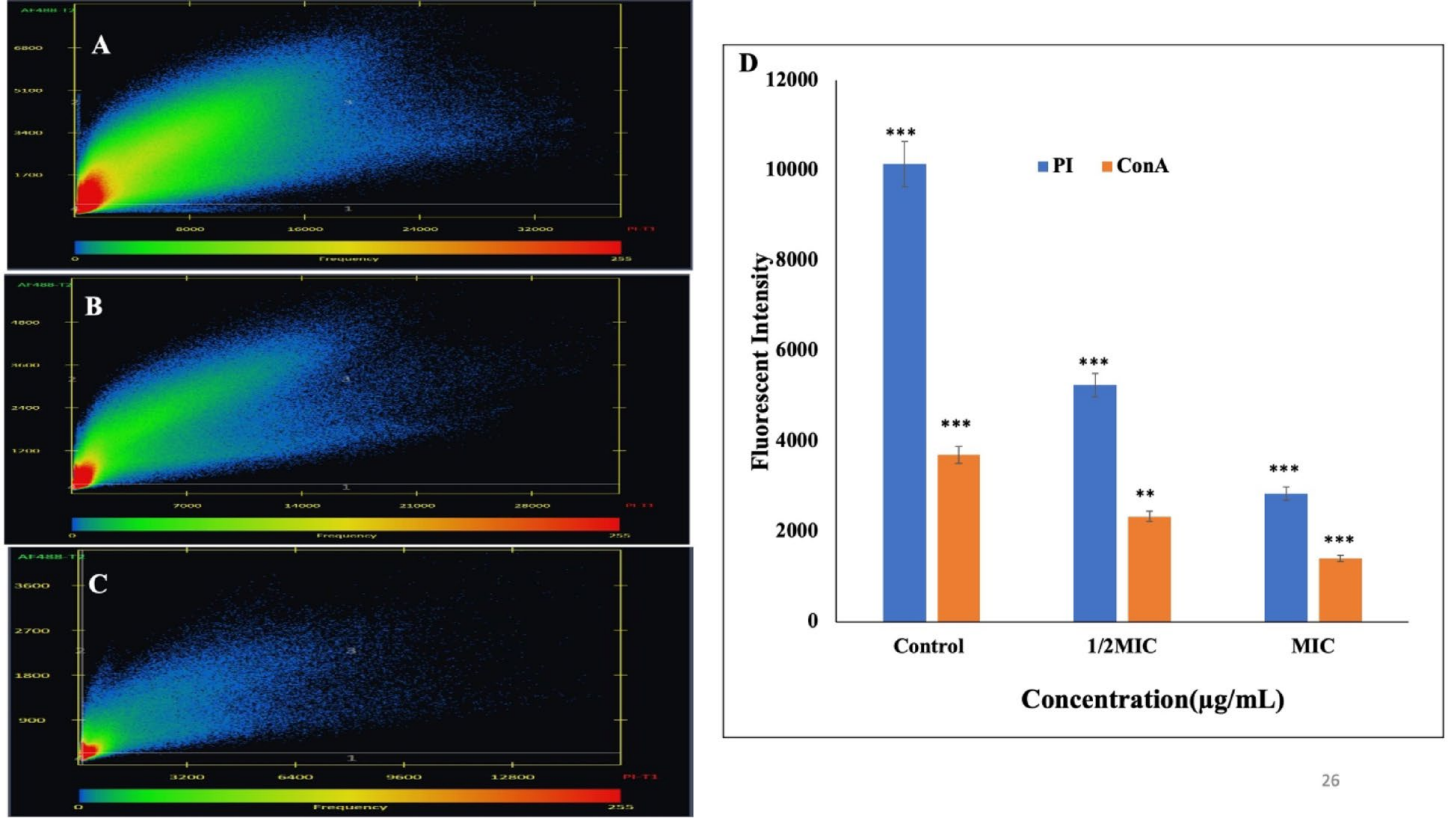
CLSM fluorescence intensity profiles of *S. marcescens* biofilms: (**A**) Control, (**B**) 1/2MIC syringic acid-treated, (**C**) MIC syringic acid-treated, and (**D**) Quantitative comparison of matrix fluorescence intensity between control and syringic acid-treated samples, indicating concentration-dependent disruption of biofilm components

Similarly, when biofilms were stained with propidium iodide (PI) to visualize the eDNA component, control samples exhibited greater red fluorescence compared to those treated with CA and SA at both 1/2MIC and MIC . This indicates that eDNA is a key structural component of the mature biofilm matrix. The interaction of both phenolic acids with eDNA and polysaccharides likely compromised the structural integrity of the biofilms, leading to their disruption (Figs. 11 and 12). Quantitative relative fluorescence analysis confirmed a concentration-dependent reduction in both polysaccharides and eDNA. As shown in the figure, untreated *S. marcescens* biofilms displayed significantly higher fluorescence than those treated with phenolic acids. Treatment with CA led to a reduction in PI fluorescence intensity by 52% at 1/2MIC and 82% at MIC, while ConA fluorescence decreased by 56% and 70%, respectively (Fig. 13). For SA, ConA intensity dropped by 37% at 1/2MIC and 62% at MIC, whereas PI fluorescence was reduced by 48% and 72%, respectively. These results demonstrate that CA and SA significantly deplete the key structural components of the biofilm matrix (Fig. 14), thereby destabilising mature biofilms. This biofilm matrix targeting activity highlights the potential of phenolic acids in enhancing the bacterial susceptibility to antimicrobial agents and biofilm-associated persistence of *S. marcescens.*

### Phenolic compounds potential to attenuate QS-induced virulence factors production

**Fig. 15:**
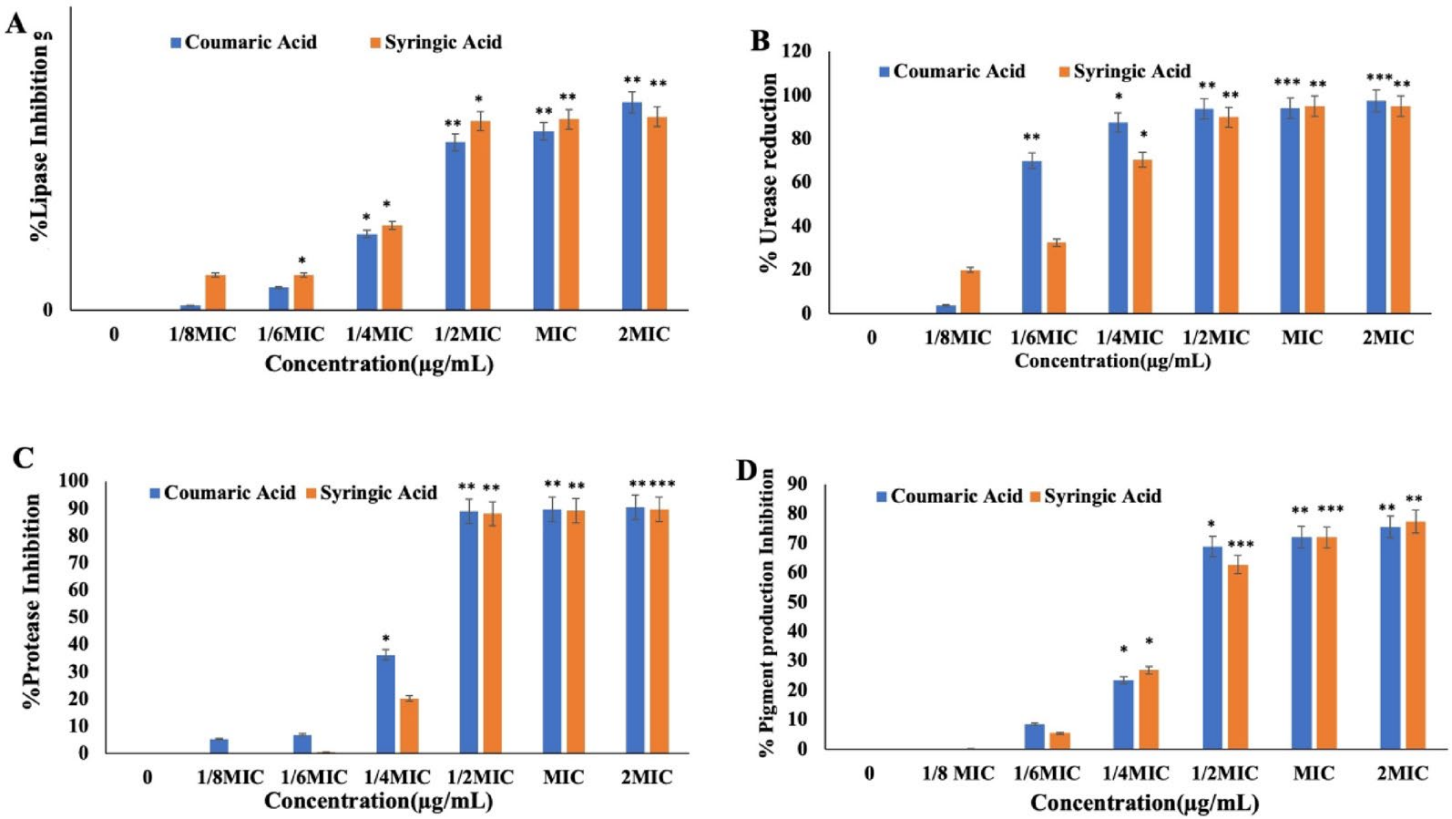
Effect of coumaric acid (CA) and syringic acid (SA) on quorum sensing (QS)-regulated virulence factor production in *S. marcescens*: (**a**) Lipase, (**b**) Urease, (**c**) Protease, and (**d**) Prodigiosin. Results represent the mean of three independent experiments. *P* < *0.05*

*S. marcescens* produces several virulence factors that contribute to infection and pathogenicity. Lipase, a virulence factor that degrades host lipids and facilitates infection spread was significantly suppressed by treatment with CA and SA, showing 61% and 57% inhibition, respectively (Fig. 15A). Urease is another key virulence factor produced by *S. marcescens*, contributing to pathogenicity by generating toxic levels of ammonia within the host. Treatment with CA and SA resulted in a substantial inhibition of urease activity, with both compounds showing approximately 97% reduction at their MICs (Fig. 15B). Protease, a major virulence factor of *S. marcescens*, plays a crucial role in host tissue damage. Treatment with CA and SA led to a marked reduction in protease activity, with both compounds achieving approximately 90% inhibition at MICs (Fig. 15C). Additionally, prodigiosin, a key pigment produced by *S. marcescens*, acts as a quorum sensing molecule essential for bacterial communication and virulence progression was significantly suppressed. CA inhibited prodigiosin production by 75% and SA by 77% at their MICs compared to the untreated control (Fig. 15D). Together, these results demonstrate that CA and SA effectively target multiple virulence factors of *S. marcescens*, thereby impairing its pathogenic potential. These findings support their use as alternative therapeutic agents against antibiotic-resistant *S. marcescens* infections.

### Effect of phenolic compounds on the swimming and swarming motility of *S. marcescens*

**Fig. 16:**
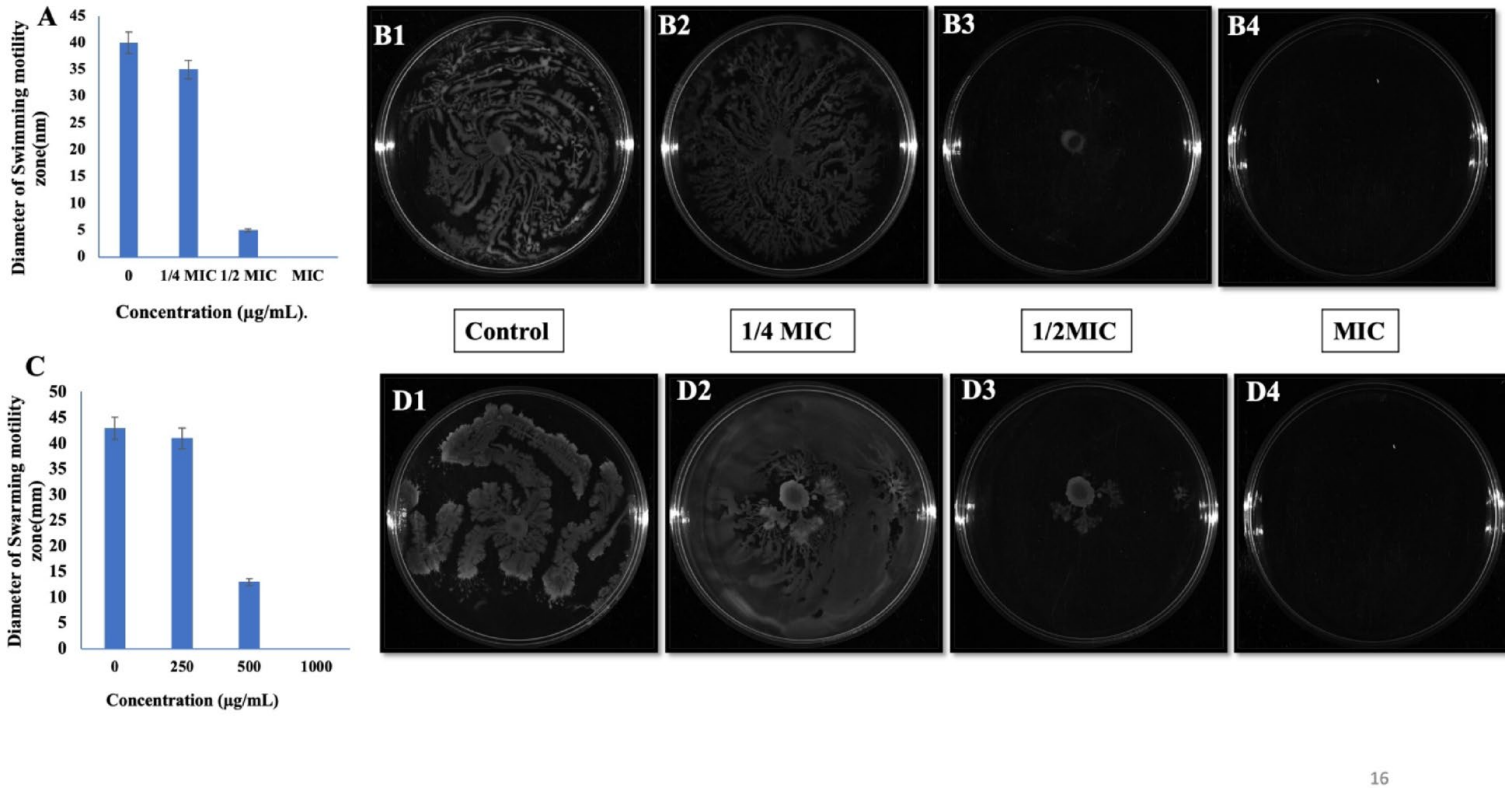
Anti-quorum sensing activity of coumaric acid (CA). (**A**) Measurement of swimming motility diameter with CA treatment. (**B1**–**B4**) Representative images showing the effect of CA on bacterial swimming motility. (**C**) Measurement of swarming motility diameter with CA treatment. (**D1**–**D4**) Representative images depicting the effect of coumaric acid on bacterial swarming motility

**Fig. 17:**
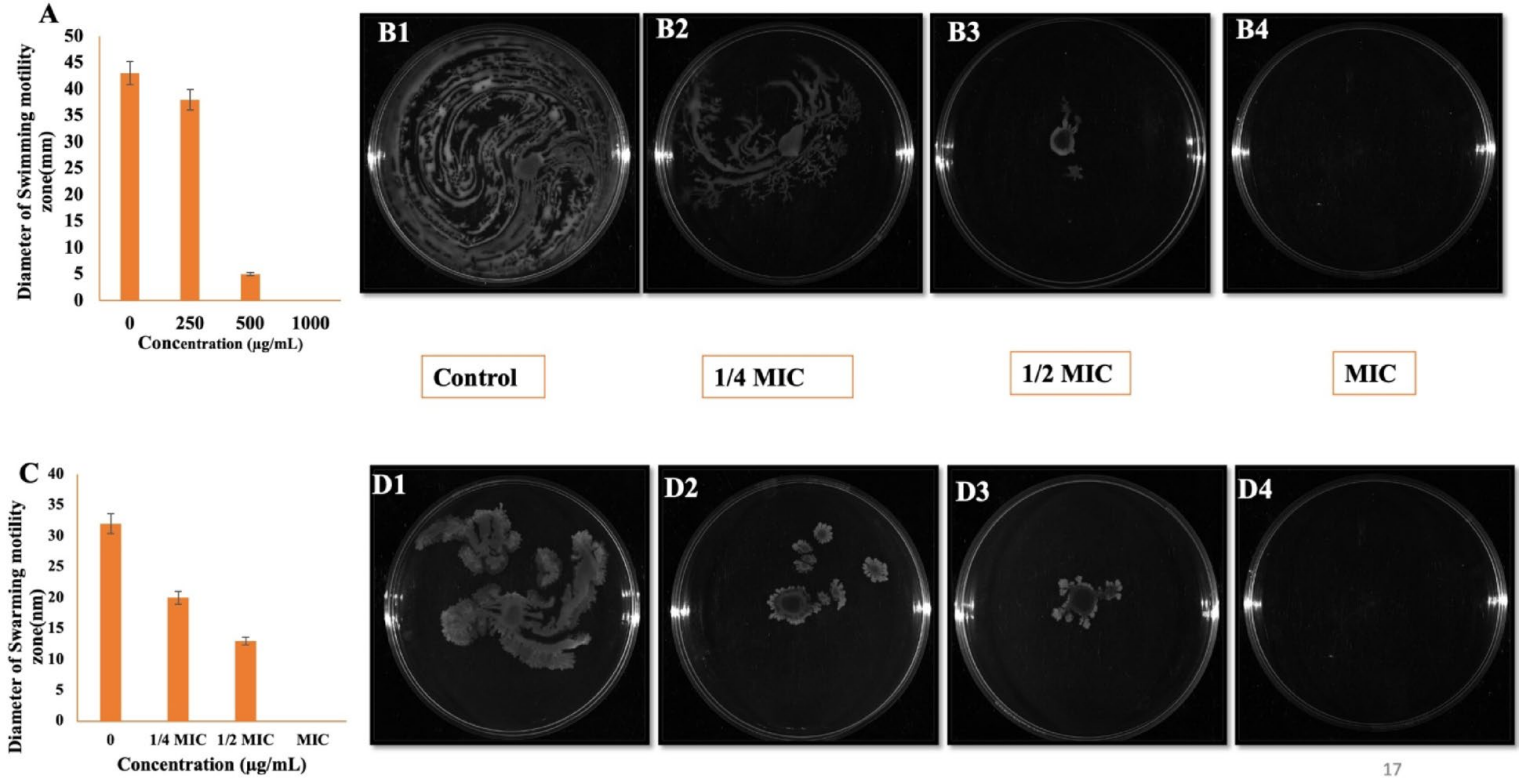
Anti-quorum sensing activity of syringic acid (SA). (**A**) Measurement of swimming motility diameter with SA treatment. (**B1**–**B4**) Representative images showing the effect of SA on bacterial swimming motility. (**C**) Measurement of swarming motility diameter with SA treatment. (**D1**–**D4**) Representative images depicting the effect of syringic acid on bacterial swarming motility

Swimming and swarming motility enable *S. marcescens* to colonize new surfaces and spread. Treatment with CA resulted in eight-fold and 3.2-fold reductions in swimming and swarming motility (Fig. 16A, C), respectively, at 1/2MIC, while motility was completely abolished at MIC (Fig. 16B, D). Similarly, SA caused 8.6-fold and 2.5-fold reductions (Fig. 17A, C), respectively, and fully abolished motility at MIC (Fig. 17 B, D). These findings indicate that CA and SA effectively disrupt the QS-regulated motility-driven colonization strategies of *S. marcescens*, thereby restricting its ability to spread and establish infection.

## Discussion

The discovery of penicillin by Alexander Fleming marked the beginning of the "golden era" of antibiotics, giving rise to the belief that infectious diseases could be eliminated through successive antibiotic innovations. However, the resurgence of infections caused by drug-resistant pathogens has challenged this optimism (Muteeb et al. 2023). Microbial adaptability has led to resistance against both existing and new antibiotics, posing a major global health threat. Methicillin-resistant *Staphylococcus aureus* (MRSA) alone causes up to 50,000 deaths annually in the US and Europe. Without effective interventions, antimicrobial resistance (AMR) is projected to cause nearly 10 million deaths annually by 2050 and cost the global economy up to $100.2 trillion (Yi et al. 2020). A key mechanism driving antibiotic resistance is biofilm formation. Biofilms are structured microbial communities embedded in an extracellular polymeric substance (EPS) matrix that enhances bacterial survival and drug tolerance. These biofilms are particularly problematic in chronic infections and those associated with medical devices. Their resilience stems from altered growth rates, gene expression, and restricted antibiotic penetration (Dincer et al. 2020). To counteract biofilm-associated resistance, plant-derived compounds have emerged as promising alternatives. Natural products from medicinal plants have demonstrated notable antibacterial and anti-biofilm properties in vitro. These compounds interfere with quorum sensing (QS) and inhibit biofilm formation through multiple mechanisms, including disruption of cell adhesion, suppression of virulence factor production, and inhibition of ECM synthesis (Tan and Vanitha 2004; Lu et al. 2014; Jagani et al. 2009).

Among these, phenolic compounds CA and SA are of particular interest due to their antioxidant and antimicrobial properties. p-Coumaric acid, a widely distributed phenolic acid, plays roles in plant physiology and human health. Syringic acid, another phenolic compound, exhibits antibacterial activity linked to its methoxy and hydroxyl functional groups, which disrupt membrane integrity and cellular processes. These phenolic acids offer a promising, natural, and environmentally sustainable strategy for combating AMR and biofilm-mediated infections (Jagani et al. 2009; Robbins 2003; Milić et al. 1998; Shahidi and Zhong 2010; Campos et al. 2009). In this current study, the antibacterial and antibiofilm potential of CA and SA against *S. marcescens* was evaluated using complex biochemical, cellular and molecular assays. The antimicrobial efficacy of the phenolic acids p-coumaric acid and syringic acid was quantitatively and qualitatively assessed, revealing potent inhibitory effects against *S. marcescens*. Notably, CA demonstrated a bactericidal mode of action, while SA functioned in a bacteriostatic capacity. Both compounds exhibited low MICs, and significantly abrogated bacterial proliferation during the logarithmic phase of growth and inhibited its colony forming ability (Fig. 1). To further delineate the mechanistic basis of their antibacterial activity, cell viability assay were performed, which demonstrated a concentration-dependent increase in membrane permeability upon treatment. This perturbation of membrane integrity suggests a direct interaction and disruption of the bacterial outer envelope architecture, suggesting that phenolic acids compromise bacterial envelope integrity and activate downstream cell death processes (Fig. 2). These findings align with previous reports demonstrating that structurally related phenolic compounds, such as 3-*p*-trans-coumaroyl-2-hydroxyquinic acid, induce membrane destabilization in Gram-negative pathogens, as confirmed by flow cytometry (Wu et al. 2016). Similarly, earlier studies have shown that essential oils (EOs) increase membrane permeability and promote leakage of intracellular components, compromising cellular integrity (Burt 2004; Sikkema et al. 1994; Tang et al. 2020). Bouyahya et al. (Bouyahya et al. 2019) and Cui et al. (Cui et al. 2018) further validated this mechanism, showing that EOs disrupt bacterial membranes, leading to efflux of proteins and macromolecules. Collectively, these findings corroborate our results.

Interestingly, Annexin V-FITC/PI staining showed that CA and SA disrupted phosphatidylserine distribution in *S. marcescens*, causing its externalization—a hallmark of apoptosis (Chakraborty et al. 2020). Treatment showed early and late apoptosis like cell death in this bacterium at MIC (Fig. 3). Similarly, earlier studies in *E. coli* demonstrated phosphatidylserine externalization, membrane depolarization, and hydroxyl radical formation under severe DNA damage (Dwyer et al. 2012; Erental et al. 2012, 2014; Choi et al. 2016). These results suggest that CA and SA disrupt membrane integrity and intracellular homeostasis, triggering programmed cell death pathways analogous to eukaryotic apoptosis. This highlights the multifaceted antibacterial mechanisms of these phenolic acids and their potential as lead compounds for next-generation antimicrobials that can overcome conventional resistance.

Biofilm cells show greater antibiotic tolerance and environmental resistance than planktonic cells, complicating eradication (Tang et al. 2020; Abdallah et al. 2015; Srey et al. 2013). Adhesion, driven by bacterial surface hydrophobicity, is the critical initial step in biofilm formation (Re et al. 2001; Aslam 2008). Our results showed that CA and SA treatment significantly reduced hydrophobicity to 70% and 80% at 1/2 MIC and MIC respectively, impairing adhesion and biofilm development, thereby promoting bacterial clearance (Fig. 4D).

As illustrated in Fig. 4A, both CA and SA exhibited significant anti-biofilm activity against *S. marcescens* at MIC and 1/2 MIC levels, with a concentration-dependent reduction in biofilm biomass. Notably, both compounds were highly effective in disrupting preformed biofilms at MIC, highlighting their therapeutic potential in preventing and managing biofilm-associated infections (Fig. 4B). These results are consistent with prior reports on the biofilm-disrupting efficacy of phytochemicals such as quercetin, myricetin, scutellarin, flavone, trans-cinnamaldehyde, and p-coumaric acid against *S. aureus* and *E. coli* (Alain et al. 2022; Pandey et al. 2024b; Kot et al. 2015).

To elucidate the antibiofilm mechanism of CA and SA, extracellular DNA (eDNA) quantification and binding assay was performed. eDNA plays a crucial role in EPS matrix crosslinking, stabilization, and biofilm maturation (Vishwakarma and Sirisha 2020; Montanaro et al. 2011). Our findings revealed a concentration-dependent degradation of eDNA upon treatment with these phenolic acids, suggesting their disruptive action on the biofilm matrix (Fig. 4E). This was further substantiated by an eDNA binding assay, wherein both CA and SA competitively displaced ethidium bromide from isolated eDNA, leading to reduced fluorescence, indicating strong binding affinity (Fig. 5). These results demonstrate that CA and SA not only hinder biofilm initiation by altering cell surface hydrophobicity but also effectively eradicate mature biofilms by targeting and degrading the eDNA scaffold, highlighting their potential as promising therapeutic agents against *S. marcescens* biofilm-associated infections.

Scanning electron microscopy (SEM) is a powerful tool for visualizing biofilm architecture and assessing the impact of antibiofilm agents due to its high-resolution imaging capabilities (Norton et al. 1998). In this study, SEM analysis revealed that both CA and SA significantly disrupted mature *S. marcescens* biofilms and induced morphological changes in bacterial cells (Fig. 6). Notably, CA treatment led to cell elongation, a characteristic response to β-lactam-induced inhibition of septum formation in Gram-negative bacteria (Vishwakarma et al. 2022; Hannig et al. 2010; Jacques et al. 1991; Gomes and Mergulhão 2017). CLSM imaging analysis corroborated these findings, showing reduced biofilm biomass and viable cells upon treatment (Figs. 7, 8, 9 and 10). The observed disruption is attributed to the degradation of eDNA, a key structural component of the biofilm matrix essential for adhesion and maturation. Reduced fluorescence intensity confirmed lower eDNA and EPS content (Figs. 11, 12, 13 and 14). Together, these results highlight the ability of CA and SA to compromise biofilm integrity and enhance bacterial susceptibility, supporting their potential as effective antibiofilm agents (Das et al. 2010; Dengler et al. 2015; Warraich et al. 2020; Karygianni et al. 2020; Fu et al. 2021).

The quorum sensing (QS) pathway regulates the expression of multiple virulence factors, facilitating bacterial adaptation and survival within the host environment (Rutherford and Bassler 2012). Targeting these virulence factors represents a viable strategy to inhibit biofilm formation and associated pathogenicity. In this study, CA and SA treatment significantly suppressed the production of key virulence determinants in *S. marcescens*, including urease (97%), lipase (61% and 57%), protease (90%), and prodigiosin (75% and 77%) (Fig. 8). These findings are consistent with previous reports where plant-derived compounds such as quercetin, ferulic acid, gallic acid, coumaric acid, and syringic acid attenuated QS-regulated virulence in *P. aeruginosa*, *S. aureus*, *K. pneumoniae*, and *S. epidermidis* (Meena et al. 2021; Pattnaik et al. 2018; Lin et al. 2022; Minich et al. 2022). Additionally, fthese phenolic acids completely inhibited both swimming and swarming motility in *S. marcescens*, further implicating interference with the QS system and underscoring its potential to disrupt biofilm development and disease progression.

## Conclusion

This study explored the antimicrobial potential of natural phenolic compounds—coumaric acid and syringic acid—against *Serratia marcescens*. These compounds induced bacterial membrane permeabilization, ultimately leading to cell death, and notably triggered externalization of phosphatidylserine, indicative of apoptosis-like death. They exhibited a dose-dependent inhibition of biofilm formation and effectively disrupted mature biofilms by targeting the eDNA component of the extracellular polymeric substance (EPS) matrix. Additionally, both compounds attenuated the quorum sensing (QS) pathway, as evidenced by the suppressed production of key virulence factors and inhibition of bacterial motility (swimming and swarming). Collectively, these findings highlight the potential of coumaric and syringic acids as promising antibiofilm agents for combating *S. marcescens* biofilm-associated infections.

## Supplementary Information

Below is the link to the electronic supplementary material.

Supplementary file 1: Fig.1. Structure of phenolic compounds (A) Coumaric Acid (B) Syringic Acid

## Data Availability

The data is available from the corresponding author upon reasonable request.
