# Supplementary figures and images for: Deciphering the mechanisms of antibacterial and antibiofilm potential of phenolic compounds against Serratia marcescens
Source: Bioresour Bioprocess. 2025 Dec 8;12(1):147. doi: 10.1186/s40643-025-00988-0 (PMC12686276; doi:10.1186/s40643-025-00988-0)

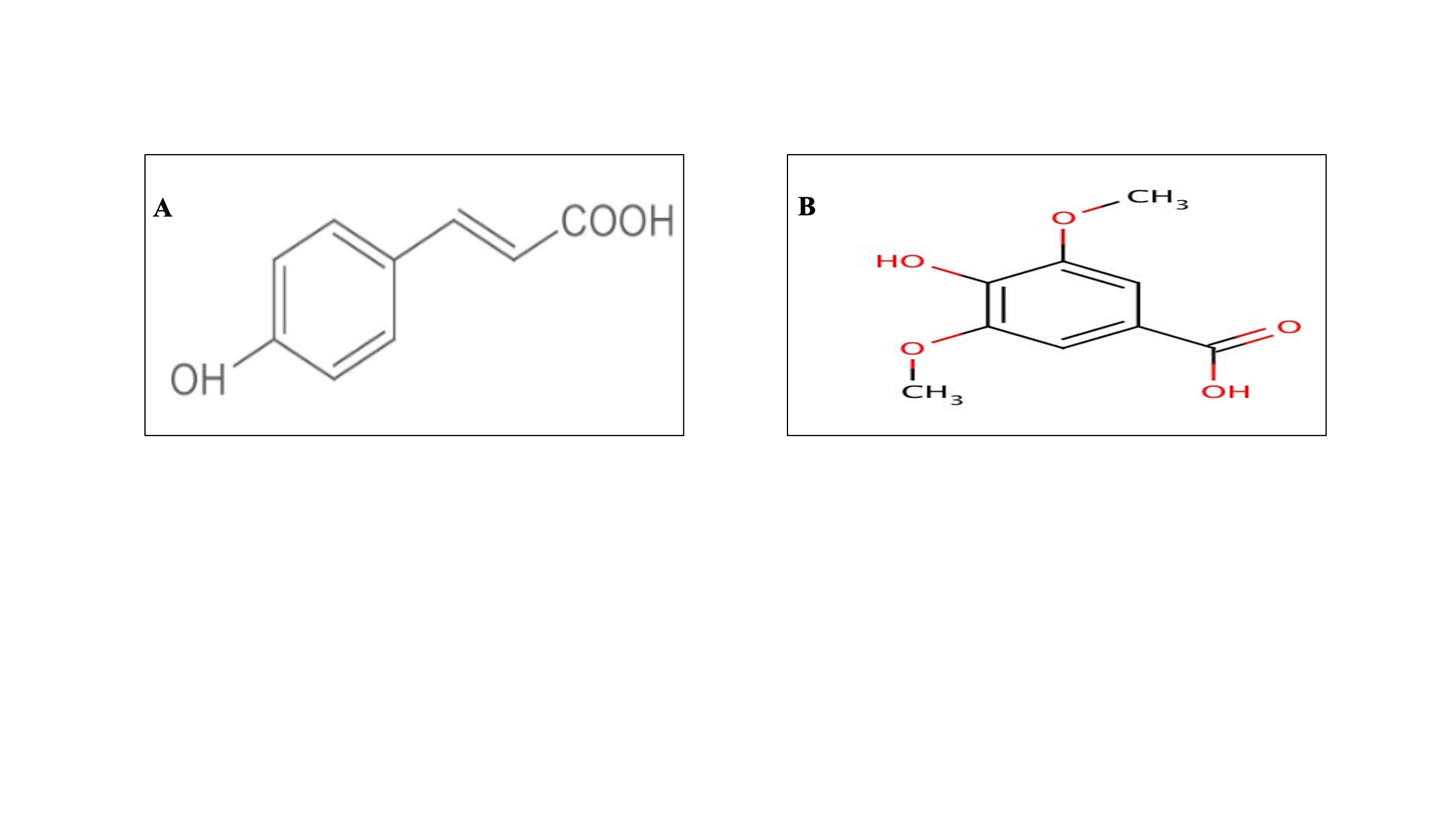

Supplement: Supplementary file 1 — Fig.1. Structure of phenolic compounds (A) Coumaric Acid (B) Syringic Acid [file 40643_2025_988_MOESM1_ESM.jpg]
